# The oncogenic role of circPVT1 in head and neck squamous cell carcinoma is mediated through the mutant p53/YAP/TEAD transcription-competent complex

**DOI:** 10.1186/s13059-017-1368-y

**Published:** 2017-12-20

**Authors:** Lorena Verduci, Maria Ferraiuolo, Andrea Sacconi, Federica Ganci, Jlenia Vitale, Teresa Colombo, Paola Paci, Sabrina Strano, Giuseppe Macino, Nikolaus Rajewsky, Giovanni Blandino

**Affiliations:** 1 Oncogenomic and Epigenetic Unit, Italian National Cancer Institute, “Regina Elena”, 00144 Rome, Italy; 2 Molecular Chemoprevention Unit, Italian National Cancer Institute, “Regina Elena”, 00144 Rome, Italy; 3 Institute for Computing Applications “Mauro Picone”, National Research Council, 00185 Rome, Italy; 4 Institute for System Analysis and Computer Science “Antonio Ruberti”, National Research Council, 00185 Rome, Italy; 5 Department of Cellular Biotechnologies and Hematology, La Sapienza University of Rome, 00161 Rome, Italy; 6 Systems Biology of Gene Regulatory Elements, Berlin Institute for Medical Systems Biology, Max Delbrück Center for Molecular Medicine, D-13125 Berlin, Germany

**Keywords:** circPVT1, mut-p53, PVT1, YAP, TEAD, miR-497-5p, HNSCC

## Abstract

**Background:**

Circular RNAs are a class of endogenous RNAs with various functions in eukaryotic cells. Worthy of note, circular RNAs play a critical role in cancer. Currently, nothing is known about their role in head and neck squamous cell carcinoma (HNSCC). The identification of circular RNAs in HNSCC might become useful for diagnostic and therapeutic strategies in HNSCC.

**Results:**

Using samples from 115 HNSCC patients, we find that circPVT1 is over-expressed in tumors compared to matched non-tumoral tissues, with particular enrichment in patients with TP53 mutations. circPVT1 up- and down-regulation determine, respectively, an increase and a reduction of the malignant phenotype in HNSCC cell lines. We show that circPVT1 expression is transcriptionally enhanced by the mut-p53/YAP/TEAD complex. circPVT1 acts as an oncogene modulating the expression of miR-497-5p and genes involved in the control of cell proliferation.

**Conclusions:**

This study shows the oncogenic role of circPVT1 in HNSCC, extending current knowledge about the role of circular RNAs in cancer.

**Electronic supplementary material:**

The online version of this article (doi:10.1186/s13059-017-1368-y) contains supplementary material, which is available to authorized users.

## Background

Circular RNAs (circRNAs) are a class of endogenous RNAs that have been known for more than a decade [1]. Original hypotheses on circRNAs ascribed them the role of plant viroids [2] and Hepatitis delta virus molecules [3] or considered them the result of transcriptional noise [4]. More recently, circRNAs have emerged for their potential functions in the regulation of gene expression [5].

CircRNAs are mostly generated from coding or noncoding exons, but they can also derive from intronic, antisense, 5′ or 3′ untranslated, and intergenic genomic regions [6]. Very high and conserved circRNA expression is typically found in neural tissues [7], muscle, and some hematopoietic cells, for hundreds of circRNAs [8, 9].

CircRNAs comprised of exonic sequences are produced by a poorly characterized mechanism called “back-splicing”, where a downstream 5′ splice site is joined to an upstream 3′ splice site, involving single or multiple exons [5, 10–14]. Due to lack of accessible ends, circRNAs are resistant to exonucleases and, as a result, they are more stable than linear RNA isoforms. Despite advancements in the study of circRNAs, their function in eukaryotes is not clear. According to the recent literature, they may regulate alternative splicing [10, 14], bind and sequester RNA-binding proteins and ribonucleoprotein complexes [6, 15], be translated, bind *in trans* to other RNA sequences [5], or regulate miRNA expression [6, 15].

Of particular interest is the recently discovered role of circRNAs in cancer [16–18]. In the same line, our work investigates the role of a human circRNA, circPVT1, in head and neck squamous cell carcinoma (HNSCC). CircPVT1 was first identified as circ6 by Memczak et al. [6] and then named circPVT1 after its host gene PVT1 in subsequent work [19, 20]. The PVT1 gene is frequently up-regulated in many types of cancers, including HNSCC [21–25]. The circPVT1 locus is embedded in the long non-coding RNA PVT1 and it originates from exon 2 of the PVT1 gene (human genome GRch38/hg38).

HNSCC is the sixth leading cancer by incidence worldwide and the eighth most common cause of cancer death [26, 27]. Although in the past two decades new surgical and medical treatments have improved the quality of life of patients [28–30], the 5-year survival rate is achieved by only 40–50% of patients [26].

We started our study investigating the oncogenic role of circPVT1 in HNSCC using a robust collection of human tissue samples. circPVT1 was found significantly up-regulated in tumors compared with matched non-tumoral tissues. More importantly, we have discovered that circPVT1 expression was enriched in tumors carrying mutant p53 proteins (mut-p53). Genomic data have shown that p53 is the most frequent mutated gene in HNSCC; indeed it is mutated in up to 85% of HNSCC cases and these involve mainly exons 5–8 [31–34]. We recently reported that mut-p53 cooperates with the transcriptional co-factor YAP (Yes-Associated Protein) in breast cancer cell lines [35]. YAP as an oncogene acts as an effector of the Hippo pathway, playing a critical role in the initiation and progression of several human cancers, including HNSCC [36–39]. YAP and mut-p53 proteins are able to physically interact and share a common set of transcriptional programs in cancer [35]. In our study, we found that the circPVT1 was regulated through the mut-p53/YAP/TEAD complex via its regulatory region. Moreover, our data show that circPVT1 was able to regulate its own expression through binding YAP. To date, the role of circRNAs in HNSCC is unexplored. Collectively, these findings mirror a novel alteration in the circRNA network that might contribute to the fine deciphering of the tumorigenesis occurring in mut-p53 HNSSC patients.

## Results

### circPVT1 is up-regulated in HNSCC patients with TP53 mutations

Previous studies have shown that PVT1 resides in the well-known cancer risk region 8q24 and is amplified in HNSCC [21–25]. To analyze in detail the PVT1 amplification, we used the HNSCC cancer data set provided by The Cancer Genome Atlas (TCGA) [33].

**Fig. 1:**
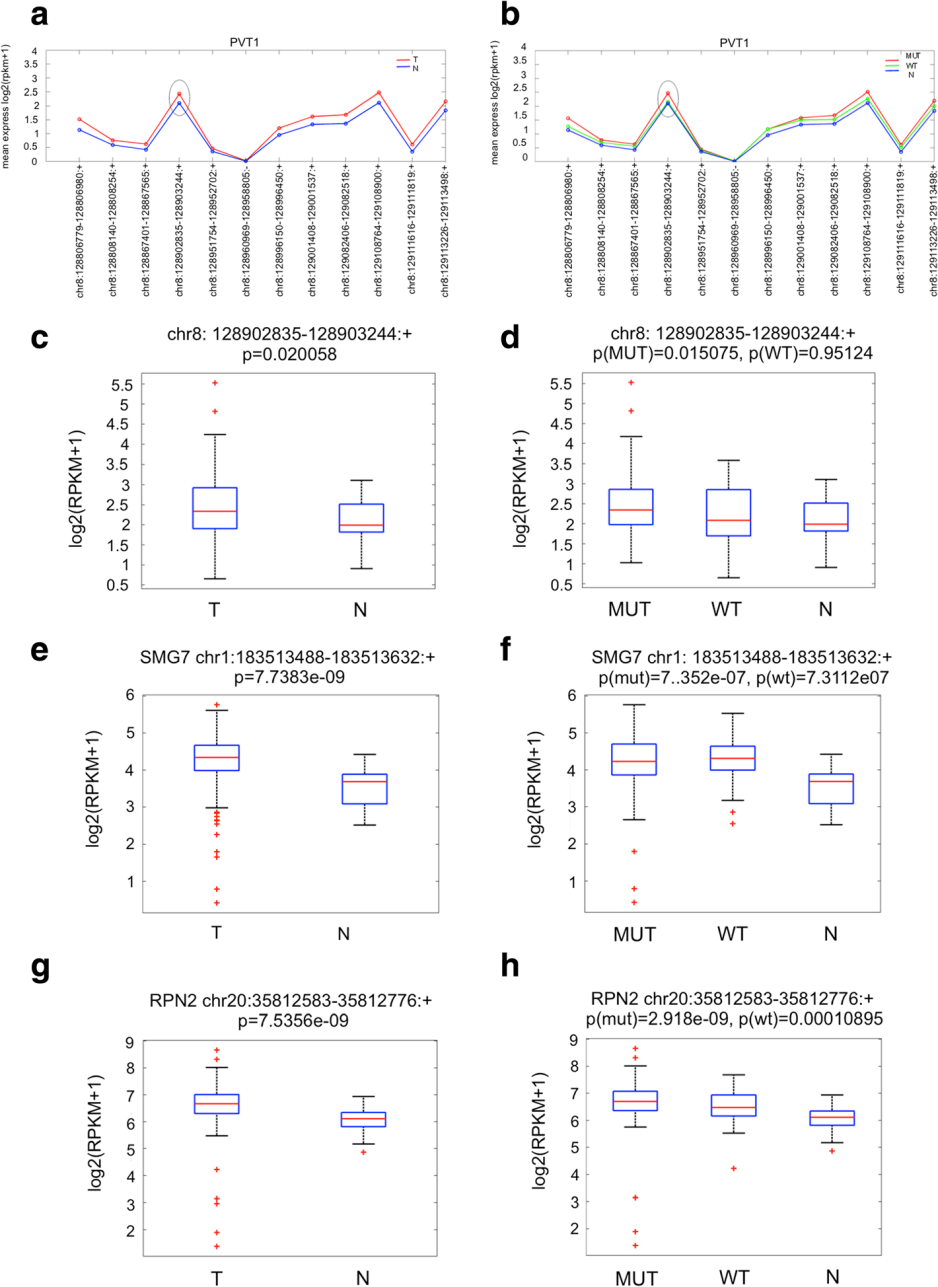
circPVT1, SMG7, and RPN2 expression in the HNSCC cancer data set. **a**–**d** Bioinformatic analysis of PVT1 and circPVT1 amplification using the HNSCC cancer data set provided by The Cancer Genome Atlas. **a** Chromosomal intervals of the PVT1 gene in tumor and non-tumoral samples. The peak related to circPVT1 is indicated by an *oval* (Wilcoxon rank sum test, *p* < 0.05). **b** Chromosomal intervals of the PVT1 gene in tumor samples (divided into mut-p53 and wt-p53) and in non-tumoral samples. The peak related to circPVT1 is indicated by an *oval* (Wilcoxon rank sum test, *p* < 0.05). **c** Chromosome interval containing circPVT1 in tumor and non-tumoral samples. **d** Chromosome interval containing circPVT1 in tumor (divided into mut-p53 and wt-p53) and non-tumoral samples. **e** Chromosome interval containing SMG7 in tumor and non-tumoral samples. **f** Chromosome interval containing SMG7 in tumor (divided into mut-p53 and wt-p53) and non-tumoral samples. **g** Chromosome interval containing RPN2 in tumor and non-tumoral samples. **h** Chromosome interval containing RPN2 in tumor (divided into mut-p53 and wt-p53) and non-tumoral samples. *MUT* p53 mutation, *N* non-tumoral, *T* tumoral, *WT* wild type

At first, we considered individually the chromosome intervals representing the PVT1 gene. We initially compared non-tumoral samples versus tumor samples (Fig. 1a). We then evaluated non-tumoral samples with either mut-p53 or wt-p53 tumor samples (Fig. 1b). Next, we focused our analysis on the chromosome interval containing circPVT1.

circPVT1 was shown to be up-regulated in tumor samples (Fig. 1a, c). Interestingly, circPVT1 was significantly up-regulated in tumors carrying TP53 mutations but not in those with intact TP53 (Fig. 1b, d). This might suggest that the presence of the mut-p53 protein accounts for circPVT1 up-regulation observed in HNSSC patients. To further investigate the specificity of the association between TP53 mutations and circPVT1 amplification we analyzed the PVT1 amplification in relation to the FAT1 and CDKN2A mutations, the second (22.46%) and the third (22.07%) most frequent gene mutated in HNSCC patients according to TCGA. Focusing on the PVT1 chromosome interval representing circPVT1, we did not observe any difference between tumor samples with either FAT1 or CDKN2A mutations and those with an intact FAT1 or CDKN2A gene, which were both up-regulated in comparison to non-tumoral samples (Additional file 1: Figure S1a, b). This might further support that the circPVT1 amplification observed in HNSCC with TP53 mutations is strictly connected to the mut-p53 status and is not generally related to any cancer mutations.

To further validate our bioinformatic approach we selected two other circRNAs deregulated in cancer, SMG7 and RPN2 [18]. Using the HNSCC cancer data, we found both circRNAs were up-regulated in the tumors compared to non-tumoral samples (Fig. 1e, g). Unlike circPVT1, we did not observe any specific up-regulation of SMG7 and RPN2 when comparing TP53 mutated versus wild-type p53 tumors (Fig. 1f, h). This indicated that TP53 mutations did not affect the deregulation of both SMG7 and RPN2 in HNSCC.

**Fig. 2:**
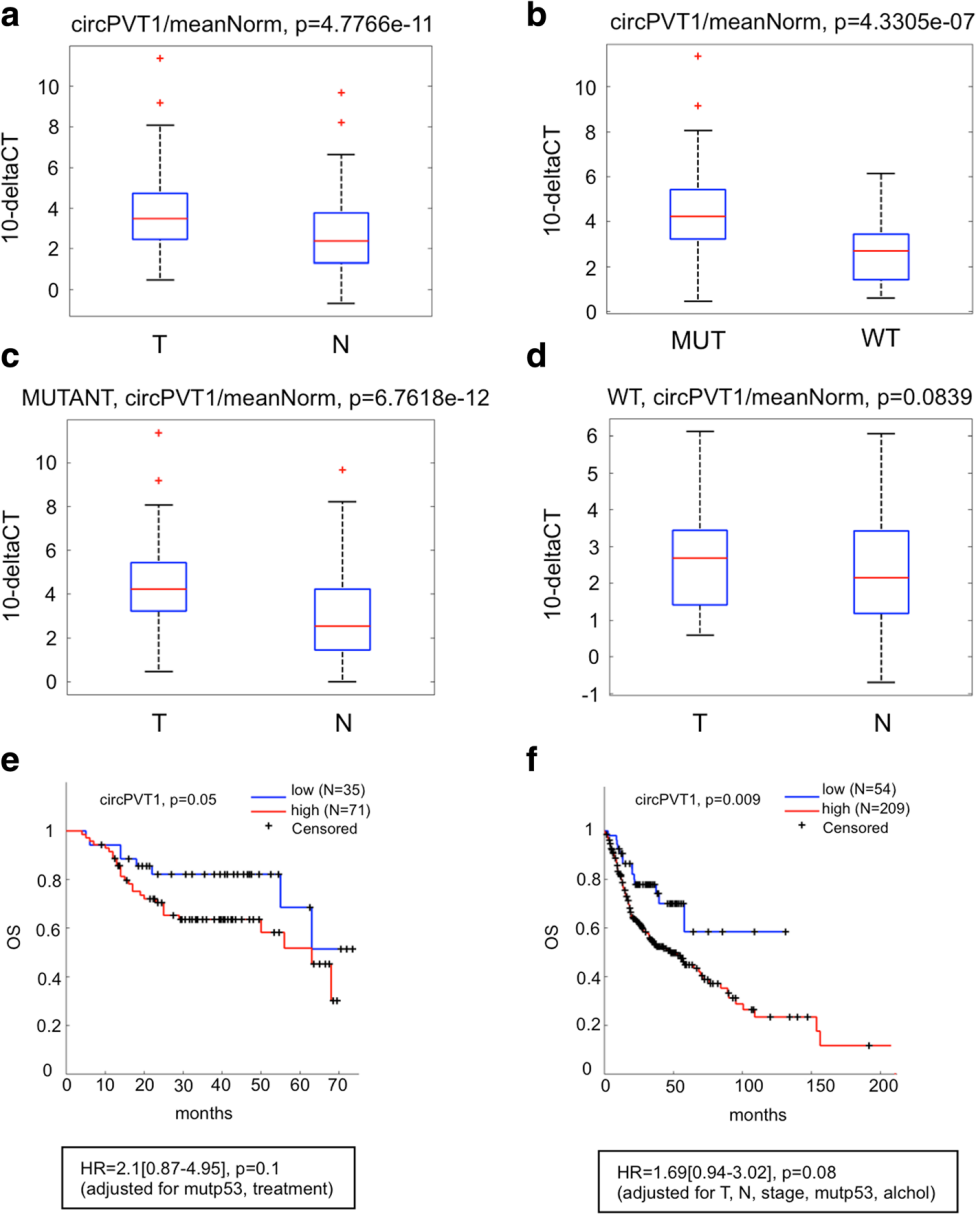
circPVT1 expression in HNSCC patients and circPVT1’s role in overall survival. **a**–**d** circPVT1 expression analysis detected by RT-qPCR. **a** circPVT1 expression analysis in tumoral samples matched with their non-tumoral counterparts. **b** circPVT1 expression analysis in mut-p53 compared to wild-type samples. **c** circPVT1 expression analysis in mutant samples divided into tumoral samples matched with their non-tumoral counterparts. **d** circPVT1 expression analysis in wild-type samples divided into tumoral samples matched with their non-tumoral counterparts. *meanNorm* mean normalizers, MUT p53 mutation, *N* non-tumoral, *T* tumoral, *WT* wild type. **e** Kaplan-Meir analysis representing the correlation between circPVT1 expression level and overall survival (*OS*) in our collection of HNSCC samples. **f** Kaplan-Meir analysis representing the correlation between circPVT1 expression level and OS in the HNSCC cancer data set. Multivariable analysis is shown at the bottom. *HR* hazard ratio

Next, the circPVT1 expression profile was assayed by real-time PCR (RT-qPCR) with divergent primers in 115 HNSCC samples and their non-tumoral counterparts [40]. Since the circRNA mechanisms of regulation are not entirely understood, we decided to normalize circPVT1 expression to the geometric mean of three different housekeeping genes as indicated in “Methods”. This approach was taken to avoid biasing our findings. circPVT1 expression was well detected in all samples used. Fig. 2a shows a statistically significant up-regulation of circPVT1 in tumor samples compared to matched non-tumoral tissues.

In previous work [40], we reported the incidence of p53 mutations in HNSCC patients used for this study, determined by direct sequencing of p53 exons 2 through 11. Of the original 121 patients used in [40], we studied here 114, adding one patient not included in [40], whose clinical characteristics are shown in Additional file 2: Table S1. In this work, 67 out of the 115 patient samples used (58%) exhibited single or multiple p53 mutations in the tumor tissue [40].

In order to determine whether the up-regulation of circPVT1 correlated with the TP53 mutations, we compared the circPVT1 expression of mut-p53 and wild-type tumoral samples. As shown in Fig. 2b, there was a statistically significant up-regulation of circPVT1 in mut-p53 compared to wild-type samples.

Furthermore, we compared circPVT1 expression between tumoral and non-tumoral samples, considering separately the mut-p53 and wild-type patients. We found that circPVT1 was significantly up-regulated only in patients with TP53 mutations, confirming the correlation between mut-p53 and circPVT1 in HNSCC (Fig. 2c, d).

The identity of circPVT1 was also analyzed by Sanger-sequencing following RT-qPCR reactions in a subgroup of patients. We selected 20 tumoral samples, including 10 wild-type and 10 mut-p53 samples. For all patients selected, we confirmed the identity of circPVT1 as shown in Additional file 1: Supplementary results 1. The list of patients ordered on the basis of circPVT1 expression and p53 status is included in Additional file 2: Table S2.

Starting from the demographic and clinical characteristics of the patients, we used a univariate linear regression analysis to test the association of circPVT1 with prognostically relevant risk factors. Considering tumor samples, we observed a significant correlation between circPVT1 and mut-p53 (Additional file 2: Table S3). This result correlates with the circPVT1 up-regulation seen in patients with TP53 mutations, as shown above. Among other factors analyzed, we also found a correlation between circPVT1 and alcohol use, with a clear tendency towards statistical significance (Additional file 2: Table S3).

We then evaluated the impact of circPVT1 on the outcomes of patients. Using the HNSCC cancer data set and our collection of HNSCC samples, we determined that patients with high circPVT1 levels had a poorer overall survival in comparison to those exhibiting low circPVT1 levels (Fig. 2e, f). Multivariable analysis confirmed that high circPVT1 levels were associated with reduced overall survival and that such association was dependent on TP53 mutations in both sample populations (Fig. 2e, f).

### circPVT1 cellular localization in HNSCC cell lines

**Fig. 3:**
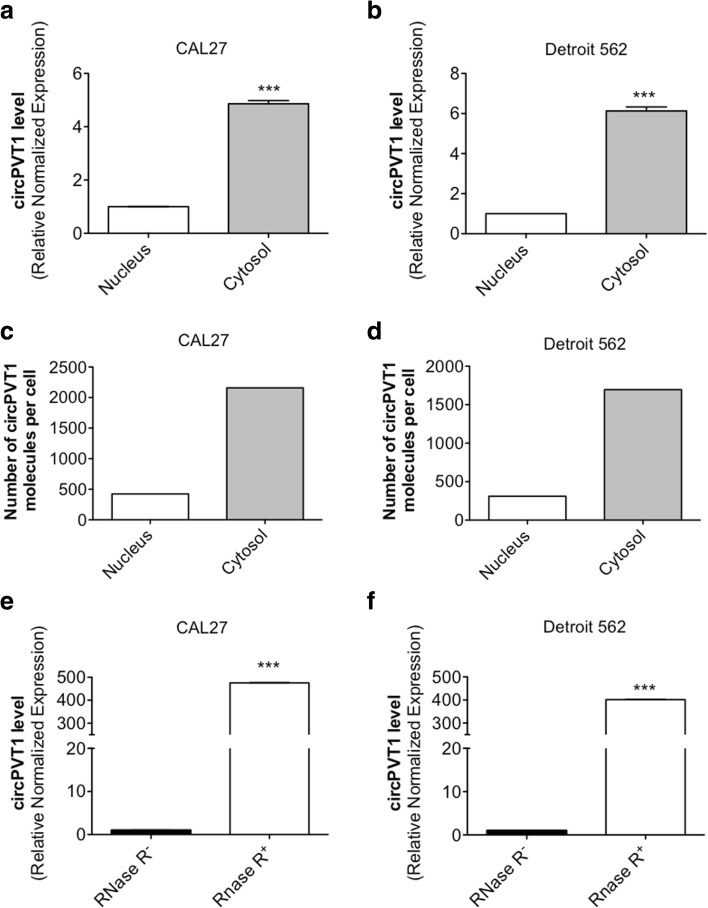
Characterization of circPVT1. **a** circPVT1 level was detected by RT-qPCR after nucleus/cytosol extraction in the CAL27 cell line. **b** circPVT1 level was detected by RT-qPCR after nucleus/cytosol extraction in the Detroit 562 cell line. **c** Number of circPVT1 molecules per cell in the CAL27 cell line. **d** Number of circPVT1 molecules per cell in the Detroit 562 cell line. **e** circPVT1 level was detected by RT-qPCR after treatment with the exonuclease RNase R in the CAL27 cell line. **f** circPVT1 level was detected by RT-qPCR after treatment with the exonuclease RNase R in the Detroit 562 cell line. Data are shown as the mean of three replicates ± standard deviation (Student’s test; *p < 0.05; **p < 0.01; ***p < 0.001)

To assess the cellular localization of circPVT1 we performed a nucleus/cytosol extraction. We found that circPVT1 was enriched in the cytoplasmic fraction but it was still present in the nucleus in both cell lines used, CAL27 and Detroit 562 (Fig. 3a, b). The CAL27 cell line is mutated for the p53 gene as a consequence of a missense mutation in codon 193 (mutp53A193T). The Detroit 562 cell line is mutated for the p53 gene as a consequence of a missense mutation in codon 175 (mutp53R175H). We estimated the approximate number of circPVT1 molecules per cell to be 425 in the nucleus and 2159 in the cytosol in the CAL27 cell line (Fig. 3c), and 310 in the nucleus and 1696 in the cytosol in the Detroit 562 cell line (Fig. 3d; details of the analysis are shown in “Methods”). A typical feature of circRNAs is their enrichment after treatment with the exonuclease RNase R, which digests linear but not circular RNA. We treated CAL27 and Detroit 562 cell lines with RNase R and measured the circPVT1 expression level in comparison to the untreated samples. For both cell lines, circPVT1 was highly enriched after the RNase R treatment (Fig. 3e, f). Collectively, these findings indicate that the circPVT1 present in HNSCC cell lines fulfils essential features of circRNAs.

### Modulation of mut-p53 expression affects circPVT1 but not PVT1 expression

**Fig. 4:**
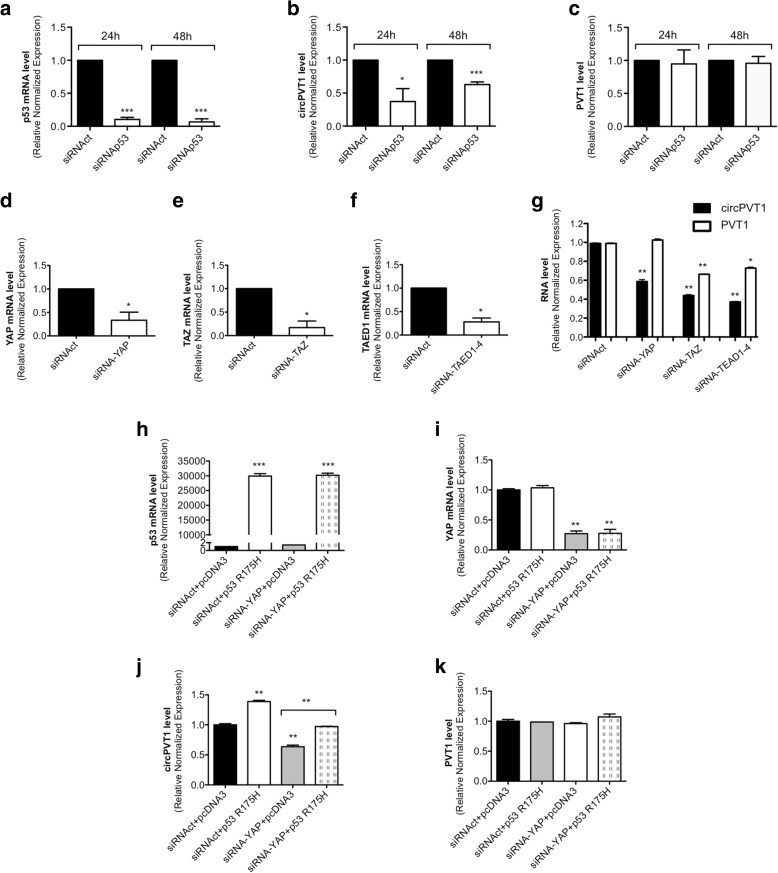
The down-regulation of mut-p53, YAP, TAZ, and TEAD affects circPVT1 but not PVT1 expression. **a**–**g** CAL27 cell line. **a** p53 mRNA level was detected by RT-qPCR 24 and 48 h after transfection with siRNAp53 or siRNAct. **b** circPVT1 level was detected by RT-qPCR 24 and 48 h after transfection with siRNAp53 or siRNAct. **c** PVT1 level was detected by RT-qPCR 24 and 48 h after transfection with siRNAp53 or siRNAct. **d** YAP mRNA level was detected by RT-qPCR 48 h after transfection with siRNA-YAP or siRNAct. **e** TAZ mRNA level was detected by RT-qPCR 48 h after transfection with siRNA-TAZ or siRNAct. **f** TEAD1 mRNA level was detected by RT-qPCR 48 h after transfection with siRNA-TEAD1-4 or in siRNAct. **g** circPVT1 and PVT1 levels were detected by RT-qPCR 48 h after transfection with siRNA-YAP, siRNA-TAZ, or siRNA-TEAD1-4. **h**–**k** A253 cell line. **h** p53 mRNA level was detected by RT-qPCR 48 h after transfection. **i** YAP mRNA level was detected by RT-qPCR 48 h after transfection. **j** circPVT1 level was detected by RT-qPCR 48 h after transfection. **k** PVT1 level was detected by RT-qPCR 48 h after transfection. The transfections were performed using the siRNAs and the vectors indicated in the graphs. Data are shown as mean of three replicates ± standard deviation (Student’s test; *p < 0.05; **p < 0.01; ***p < 0.001)

To investigate at a molecular level the relationship between circPVT1 and mut-p53 protein, we used four HNSCC cell lines as an in vitro model, CAL27, Detroit 562, FaDu, and A253. We reduced the expression level of mut-p53 by a specific siRNA (siRNAp53) in the CAL27 cell line and observed the subsequent circPVT1 expression. To assess the specificity of the siRNA against p53, we used a siRNA control (siRNAct) and analyzed the p53 expression at different time points by RT-qPCR. siRNAp53 decreased the p53 expression by about 90% at 24 and 48 h (Fig. 4a). Next, we analyzed the circPVT1 expression after down-regulation of p53. As shown in Fig. 4b, we obtained a statistically significant down-regulation of circPVT1 expression by about 60% at 24 h and 40% at 48 h. Finally, to confirm that circPVT1 functioned independently of its host gene PVT1, we evaluated the PVT1 expression level after p53 down-regulation. We did not observe any down-regulation of PVT1 (Fig. 4c), showing that circPVT1 and mut-p53 are interconnected with each other without PVT1 involvement. Moreover, we used two additional siRNAs against p53, siRNAp53 3′ UTR and siRNAp53 smart pool, to verify that the p53 down-regulation affected the circPVT1 expression (Additional file 1: Figure S2a). We obtained a statistically significant down-regulation of circPVT1 expression at 24 and 48 h with both siRNAs (Additional file 1: Figure S2b). Once again, we did not observe any down-regulation of PVT1 expression (Additional file 1: Figure S2c).

We also investigated the existence of an inverse relationship between circPVT1 and mut-p53, i.e., whether modified circPVT1 levels influence p53 expression. The p53 protein level was not affected by circPVT1 down-regulation, therefore excluding the inverse regulation (Additional file 1: Figure S2d).

We also showed that the reduction of p53 affected circPVT1 level in Detroit 562 cells. Again, siRNAp53, siRNAp53 3′ UTR, and siRNAp53 smart pool significantly reduced circPVT1 expression with no effect on PVT1 expression (Additional file 1: Figure S2e–g). As seen before, also in the Detroit 562 cell line, modified circPVT1 levels had no influence on p53 expression (data not shown).

A specific DNA binding consensus for mut-p53 protein has not been characterized so far. For this reason, we focused first on possible post-transcriptional regulation of mut-p53 on circPVT1, investigating if mut-p53 was able to bind the mature form of circPVT1. Using RNA immunoprecipitation (RIP), we evaluated the circPVT1 level in CAL27 cells after p53 immunoprecipitation using input and IgG as controls. No circPVT1 signal was detected by RT-qPCR after p53 immunoprecipitation, showing that p53 did not influence circPVT1 expression through direct binding (Additional file 1: Figure S2h).

Finally, to confirm the absence of p53 binding to circPVT1, we co-expressed mut-p53 and circPVT1 in H1299 cells, a p53-devoid human non-small cell lung carcinoma cell line (p53 null). The two vectors expressing circPVT1 were generated according to the methods described in [15, 41] (Additional file 1: Figure S2i). After the co-expression of mut-p53 and circPVT1 in H1299 cells (Additional file 1: Figure S2j-l) we performed p53 immunoprecipitation (Additional file 1: Figure S2m) and evaluated the circPVT1 level by RT-qPCR. The circPVT1 signal obtained was similar to that of the housekeeping gene GAPDH, showing the absence of direct binding of p53 to circPVT1 (Additional file 1: Figure S2n, o). Moreover, we measured the expression of four different intronic regions upstream of circPVT1, retained in the RNA population, putatively involved in its circularization and indicated as circPVT1_UP1, circPVT1_UP2, circPVT1_UP3, and circPVT1_UP4. We did not observe any significant signal, showing that p53 did not bind these regions (Additional file 1: Figure S2p-s).

### circPVT1 expression is regulated through the mut-p53/YAP/TEAD complex on its own promoter

YAP and its paralogue TAZ (Tafazzin) are the major downstream effectors of the Hippo pathway, and TEAD family proteins (TEA Domain Family Member 1) mainly mediate their biological functions [42–44]. In previous work, we showed that YAP physically interacts with mut-p53 proteins, enhancing their pro-proliferative transcriptional activity [35]. In this role, YAP was shown to have distinct functions from its paralog TAZ in relation to the oncogenic pathway involved. In fact, YAP depletion reduced the expression of cell cycle genes regulated by mut-p53 [35], while TAZ depletion did not. Since YAP has been shown to be an important co-factor of mut-p53 in cancer, we investigated the impact of the down-regulation of YAP and its partners, TAZ and the TEAD family proteins, on the expression level of circPVT1 and PVT1 (Fig. 4d–f; Additional file 1: Figure S3a–c).

YAP affected circPVT1 expression but not that of PVT1, while TAZ and the TEAD family proteins affected the expression of both circPVT1 and PVT1, in the CAL27 cell line (Fig. 4g). These data indicate that mut-p53 and YAP specifically regulated circPVT1 while TAZ and TEAD regulated both circPVT1 and its host gene PVT1.

To further verify the impact of YAP down-regulation on circPVT1 expression, we used another siRNA against YAP, siRNA-YAP2 (Additional file 1: Figure S3d). We obtained a statistically significant down-regulation of circPVT1 expression at 24 and 48 h, whereas no PVT1 down-regulation was observed (Additional file 1: Figure S3e, f).

Reduction of YAP using both siRNA-YAP and siRNA-YAP2 (Additional file 1: Figure S3g) affected circPVT1 expression also in the Detroit 562 head and neck cell line with no effect on the PVT1 expression level (Additional file 1: Figure S3h, i).

To assess whether mut-p53 and YAP finely regulate the circPVT1 expression level, we performed a rescue experiment in the A253 cell line, a human submandibular gland cell line (p53 null). After mut-p53 overexpression (mutp53R175H) in A253 cells, we observed an increase in the circPVT1 level (Fig. 4h, j). On the contrary, circPVT1 expression was reduced as a consequence of YAP down-regulation (Fig. 4i, j). As expected, circPVT1 expression was restored to control levels when YAP down-regulation was performed concomitantly with mut-p53 over-expression (Fig. 4j). These data show that mut-p53 and YAP worked together to regulate circPVT1 expression levels. No modulation of the PVT1 expression level was observed under these experimental conditions (Fig. 4k).

**Fig. 5:**
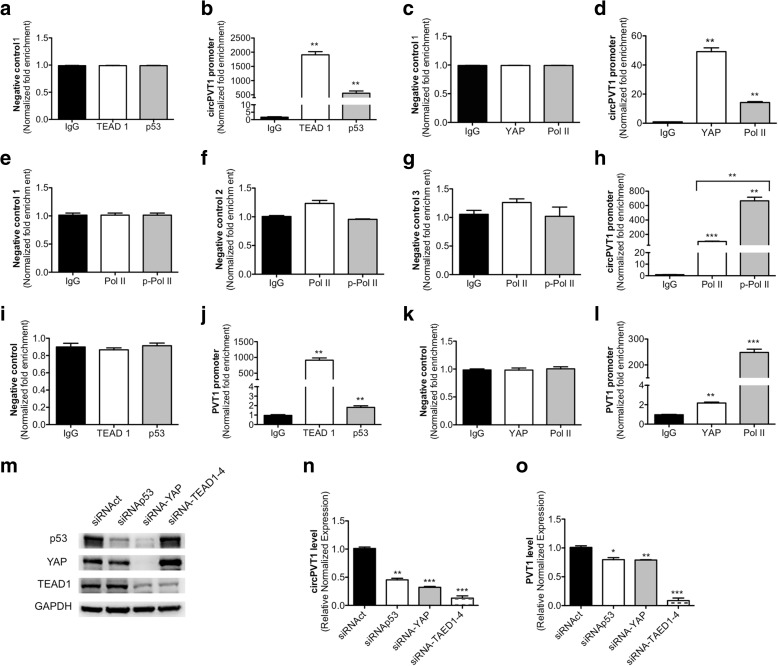
The mut-p53/YAP/TEAD complex regulates circPVT1 expression. **a**–**h** Analysis of the circPVT1 promoter. **a**, **b** ChIP analysis of p53 and TEAD1 in the CAL27 cell line. **c**, **d** ChIP analysis of YAP and Pol II in the CAL27 cell line. **e**–**h** ChIP analysis of Pol II and p-Pol II in the CAL27 cell line. **i**–**l** Analysis of the PVT1 promoter. **i**, **j** ChIP analysis of p53 and TEAD1 in the CAL27 cell line. **k**, **l** ChIP analysis of YAP and Pol II in the CAL27 cell line. **m**–**o** DRB-4sU assay. **m** p53, YAP, and TEAD1 proteins were detected by western blot after transfection of the indicated siRNA. GAPDH was used as internal loading control. **n** Nascent circPVT1 expression level was detected by RT-qPCR. **o** Nascent PVT1 expression level was detected by RT-qPCR. Data are shown as mean of three replicates ± standard deviation (Student’s test; *p < 0.05; **p < 0.01; ***p < 0.001)

In order to understand if its own promoter or the PVT1 promoter regulated circPVT1 expression, we studied the intronic region upstream of the circPVT1 start site. In particular, we studied the region upstream of exon 2, looking for TEAD consensus sequences, the cognate DNA-binding partner of YAP. We found a TEAD1 consensus binding sequence at −807 bp (ggcatcccaggg; positive strand) and a TATA box binding site at −1125 bp (gctttaaa; negative strand) from the circPVT1 start site, indicating the presence of a putative promoter region. We performed chromatin immunoprecipitation (ChIP) experiments on mut-p53 and TEAD1 in CAL27 cells to investigate whether they were able to regulate circPVT1 expression. We used a region without any TEAD1 consensus binding sequence upstream of the putative circPVT1 promoter as negative control. Mut-p53 and TEAD1 were recruited at the circPVT1 promoter containing the TEAD1 binding consensus sequence (Fig. 5a, b). Next, we investigated how YAP is involved in circPVT1 regulation at the transcriptional level by performing a ChIP assay of YAP in CAL27 cells and studying the same site of the circPVT1 promoter where mut-p53 and TEAD1 showed enrichment. We also carried out ChIP of RNA polymerase II (Pol II) to confirm that active transcription was occurring in the selected region. We found that YAP bound the circPVT1 promoter at a site containing the TEAD binding sequence, concurrently with the recruitment of Pol II (Fig. 4c, d). Additional proof that the circPVT1 promoter region is transcriptionally active was the enrichment of Pol II phosphorylated on Ser-5 of the carboxy-terminal-domain (CTD; p-Pol II). This modification is necessary to release Pol II from the initiation complex and allow it to start elongation. In this experiment, we used three negative controls, indicated as negative controls 1, 2, and 3. All the negative controls were regions without any TEAD1 consensus binding sequence and were localized upstream of the putative circPVT1 promoter. p-Pol II was strongly recruited at the circPVT1 promoter and its enrichment was higher than that of the non-phosphorylated Pol II (Fig. 5e–h). These data suggested that the mut-p53/YAP/TEAD complex regulated circPVT1 expression at the transcriptional level by residing on the circRNA promoter. We identified the PVT1 promoter region containing a TATA box binding site at −821 bp from the PVT1 start site (tgcataaacc; negative strand) and three TEAD1 consensus binding sequences at −377 bp (agctttccacgg; negative strand),−445 bp (cactttcctgc, negative strand), and −456 bp (cgccttcctcag; positive strand) from the PVT1 start site, upstream of exon 1. We performed a ChIP experiment in order to analyze the role played by TEAD1, mut-p53, and YAP on the PVT1 promoter. We used as negative control a region without any TEAD1 consensus binding sequence, upstream of the putative PVT1 promoter. All the members of the mut-p53/YAP/TEAD complex, TEAD1 (Fig. 5i, j), p53 (Fig. 5i, j), YAP (Fig. 5 k, l ), as well as Pol II (Fig. 5 k, l ), were recruited at the PVT1 promoter region containing the TEAD1 binding consensus sequence.

In order to thoroughly determine how circPVT1 is transcriptionally regulated, we used the metabolic tagging of newly transcribed RNAs by 4-thiouridine (4sU) after 5,6-dichlorobenzimidazole 1-β-D-ribofuranoside (DRB) treatment to monitor the nascent circRNA following the depletion of mut-p53, YAP, and TEAD1 proteins (Fig. 5m). We found that reduced expression of YAP, mut-p53, and TEAD1 decreased nascent circPVT1 expression when compared to control cells (Fig. 5n). The concomitant analysis of PVT1 expression confirmed that TEAD1 impacted on expression of both circPVT1 and its host gene PVT1, while a slight effect was observed on PVT1 transcription upon depletion of YAP and mut-p53 protein expression (Fig. 5o).

In aggregate, these data show that the newly identified circPVT1 promoter is a transcriptionally active region, distinct from the promoter of its host gene PVT1, and regulated by the mut-p53/YAP/TEAD complex. The PVT1 promoter also exhibited transcriptional control of circPVT1 expression.

### circPVT1 autoregulates its own expression via YAP binding

**Fig. 6:**
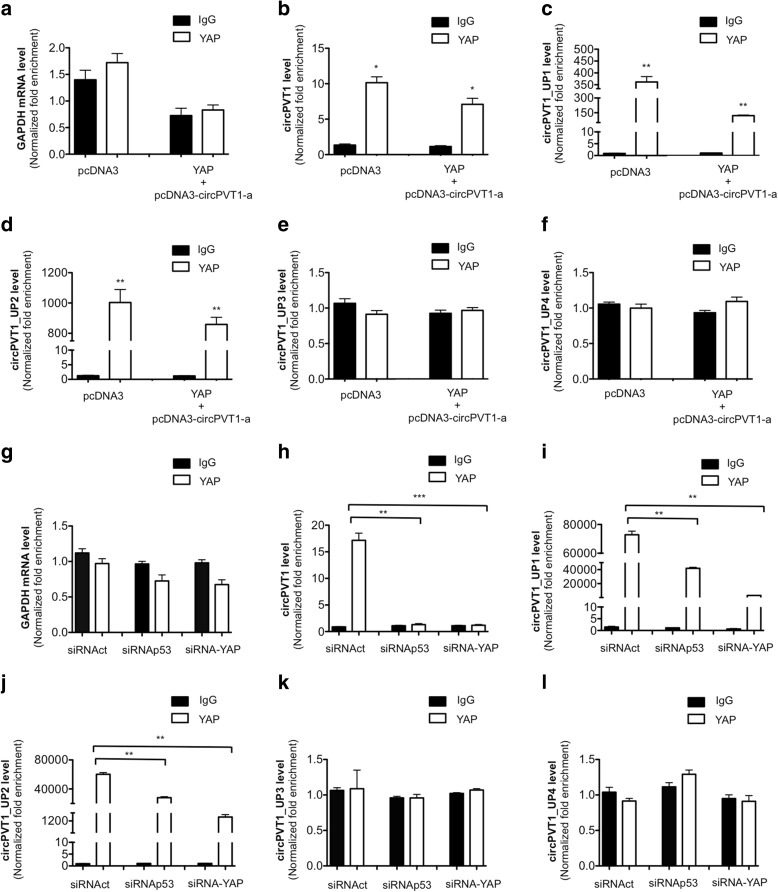
YAP binds circPVT1. **a**–**f** RIP analysis after the transfection of YAP + pcDNA-circPVT1a in the CAL27 cell line. **g**–**l** RIP analysis after the transfection of siRNAp53 or siRNA-YAP in the CAL27 cell line. GAPDH, circPVT1, circPVT1_UP1, circPVT1_UP2, circPVT1_UP3, and circPVT1_UP4 levels were analyzed by RT-qPCR. Normalization was performed to the amount of input RNA. Data are shown as mean of three replicates ± standard deviation (Student’s test; *p < 0.05; **p < 0.01; ***p < 0.001)

To understand if YAP regulates circPVT1 at the post-transcriptional level, we performed a RIP assay to verify the direct binding of the YAP protein to circPVT1. To this end, ectopic expression of YAP and circPVT1 was performed in CAL27 cells. We also tested the binding of YAP to the four different intronic regions localized upstream of the circPVT1 transcription start site, indicated as circPVT1_UP1, circPVT1_UP2, circPVT1_UP3, and circPVT1_UP4. Firstly, we confirmed the overexpression of YAP and circPVT1 (Additional file 1: Figure S4a, b) as well as the absence of modulation for PVT1 (Additional file 1: Figure S4c). The immunoprecipitation of YAP after ectopic expression of YAP and circPVT1 was confirmed as shown in Additional file 1: Figure S4d. For controls we used CAL27 cells transfected with pcDNA3 vector. Our data show that YAP, either at the endogenous level or ectopically expressed together with circPVT1, was able to bind to circPVT1 (Fig. 6a, b) and to two of the four regions up-stream of circPVT1, in particular the two regions closest to the circPVT1 transcription start site, circPVT1_UP1 and circPVT1_UP2 (Fig. 6c, d). YAP did not bind to the other two regions (circPVT1_UP3 and circPVT1_UP4) included in the analysis (Fig. 6e, f).

To dissect further the role of YAP in the regulation of circPVT1 at the post-transcriptional level, and the role of mut-p53 in this regulation, we performed a RIP assay after the transfection of siRNA-YAP and siRNAp53 separately, in CAL27 cells. The down-regulation of YAP and mut-p53 expression was confirmed as shown in Additional file 1: Figure S4e–g. The immunoprecipitation of YAP after the down-regulation of YAP and mut-p53 was confirmed as shown in Additional file 1: Figure S4h. Upon YAP down-regulation the binding to circPVT1 was lost (Fig. 6g, h). Moreover, YAP did not bind circPVT1 also as a consequence of mut-p53 down-regulation (Fig. 6g, h). These results highlight the role of mut-p53 protein in the stabilization of the YAP/circPVT1 complex. We also tested the binding of YAP to the four intronic regions, circPVT1_UP1, circPVT1_UP2, circPVT1_UP3, and circPVT1_UP4. We found that the two closest regions to the circPVT1 transcription start site, circPVT1_UP1 and circPVT1_UP2, were involved in YAP binding and were affected by YAP down-regulation (Fig. 6i, j). Although to a lesser extent than that of circPVT1, these regions were also affected by p53 down-regulation (Fig. 6i, j). The other two regions included in the analysis, circPVT1_UP3 and circPVT1_UP4, were not affected by YAP or p53 down-regulation (Fig. 6k, l).

RIP assays also revealed that the nuclear co-factor YAP bound to the mature circPVT1 and that mut-p53 had a role in the stabilization of the YAP/circPVT1 complex. These data show the presence of an operating circPVT1 in the nucleus, suggesting the capability to regulate its own expression.

**Fig. 7:**
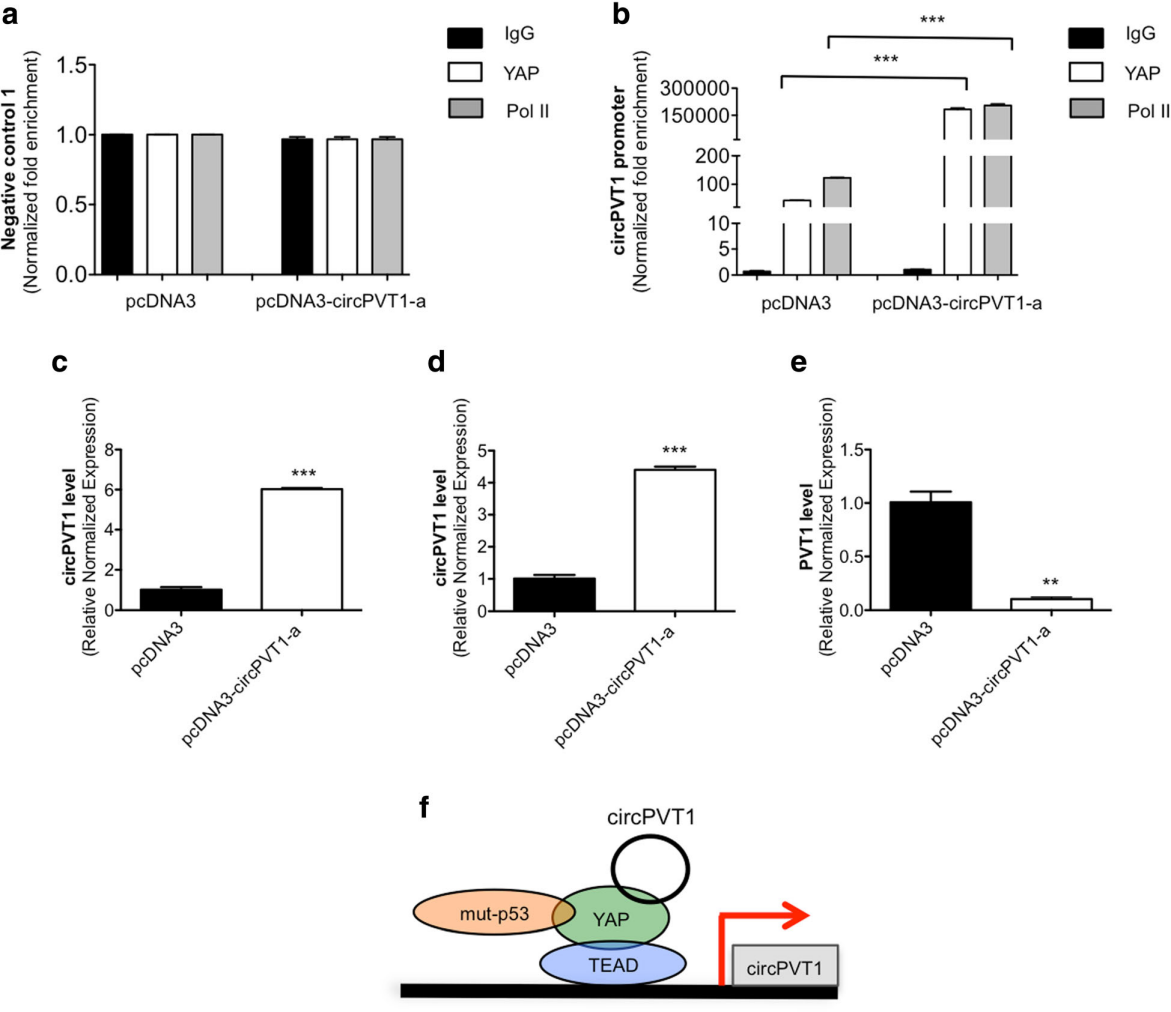
circPVT1 regulates its own expression binding the mut-p53/YAP/TEAD complex. **a**, **b** ChIP analysis of YAP and Pol II in the CAL27 cell line after the transfection of pcDNA3-circPVT1-a and pcDNA3. **c**–**e** DRB-4sU assay. **c** circPVT1 level was detected by RT-qPCR in pcDNA3-circPVT1-a and pcDNA3 transfected cells. **d** Nascent circPVT1 expression level was detected by RT-qPCR. **e** Nascent PVT1 expression level was detected by RT-qPCR. **f** The transcriptional mut-p53/YAP-circPVT1/TEAD complex bound to the circPVT1 promoter. Data are shown as mean of three replicates ± standard deviation (Student’s test; *p < 0.05; **p < 0.01; ***p < 0.001)

To test this hypothesis, we first performed ChIP assay experiments for YAP and Pol II in CAL 27 cells after circPVT1 over-expression using the pcDNA3-circPVT1-a vector. We found that both YAP and Pol II were recruited to the endogenous genomic site containing the TEAD binding sequence described above, and this recruitment was higher in the circPVT1 over-expressing condition compared to the control (Fig. 7a, b). Next, we performed the DRB-4sU assay in circPVT1 over-expression conditions. As a consequence of circPVT1 overexpression (Fig. 7c), we observed an increase in nascent circPVT1 production (Fig. 7d). Interestingly, we observed in the same experiment a reduction of the nascent PVT1 (Fig. 7e). This effect showed that, in circPVT1 over-expression conditions, the transcriptional machinery was preferentially enrolled in circPVT1 production rather than in PVT1 production. These data, added to the results of the ChIP assay after circPVT1 over-expression, confirm that circPVT1 might act within a positive self-regulatory loop, controlling and enhancing its own expression in the nucleus (Fig. 7f).

### circPVT1 modulation affects the oncogenic phenotype of HNSCC cell lines

**Fig. 8:**
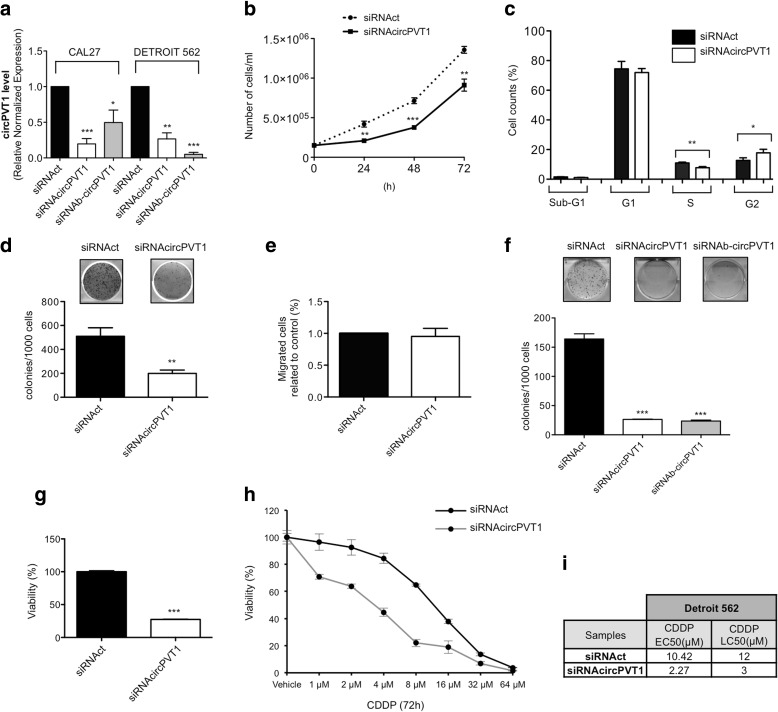
CAL27 and Detroit 562 phenotypes after down-regulation of circPVT1. **a** circPVT1 level was detected by RT-qPCR in siRNAcircPVT1, siRNAb-circPVT1, and siRNAct transfected cells. **b**–**e** CAL27 cell line. **b** Cell proliferation was determined by cell counting 24, 48, and 72 h after transfection in siRNAcircPVT1 and siRNAct transfected cells. **c** Propidium iodide flow cytometric assay to analyze the cell cycle in siRNAcircPVT1 and siRNAct transfected cells. **d** Colony formation assay in siRNAcircPVT1 and siRNAct transfected cells. *Top*: representative colony-forming assay. *Bottom*: quantification of three independent experiments by colony counting. **e** Migration assay in siRNAcircPVT1 and siRNAct transfected cells. **f**–**i** Detroit 562 cell line. **f** Colony formation assay in siRNAcircPVT1, siRNAb-circPVT1, and siRNAct transfected cells. *Top*: representative colony-forming assay. *Bottom*: quantification of three independent experiments by colony counting. **g** Viability of siRNAcircPVT1 and siRNAct transfected cells. **h** Viability after cisplatin (CDDP) treatment in siRNAcircPVT1 and siRNAct transfected cells. **i** The EC50 and the LD50 were determined using the Compusyn Software [58]. *CDDP* cisplatin, *EC50* half maximum effective concentration; *LC50* half lethal concentration. Data are shown as mean of three replicates ± standard deviation (Student’s test; *p < 0.05; **p < 0.01; ***p < 0.001)

To understand if circPVT1 down-regulation impacts the cancer cell phenotype we used two different siRNAs against circPVT1, siRNAcircPVT1 and siRNAb-circPVT1. Both siRNAs included a part of the junctional sequence of the circular RNA, and both were able to reduce significantly circPVT1 expression in CAL27 cells (Fig. 8a), with no influence on PVT1 expression (Additional file 1: Figure S5a). To assess the specificity of the siRNAs against circPVT1, we used a control siRNA (siRNAct), which did not have homology to any human gene. Compared to the control, siRNAcircPVT1 and siRNAb-circPVT1 decreased circPVT1 expression by about 80 and 50%, respectively (Fig. 8a).

We further demonstrated the specificity of the siRNAs against circPVT1 using four mismatched control siRNAs, generated according to the methods described in [45]. Two mismatched control siRNAs were designed from the sequence of siRNAcircPVT1, designated siRNAct1 and siRNAct2; the other two mismatched control siRNAs were designed from the sequence of siRNAb-circPVT1, designated siRNAb-ct1 and siRNAb-ct2. We tested these siRNAs in CAL27 and Detroit 562 cells. siRNAct1 and siRNAct2 had no effect on the circPVT1 expression level in comparison to siRNAcircPVT1, which had an effect in both CAL27 and Detroit 562 cells (Additional file 1: Figure S5b, c). Similar results were obtained for siRNAb-ct1 and siRNAb-ct2 when these siRNAs were compared to siRNAb-circPVT1 (Additional file 1: Figure S5d, e). We conclude that the mismatched control siRNAs behaved similarly to siRNAct in CAL27 and Detroit 562 cell lines.

Down-regulation of circPVT1 significantly reduced the proliferation rate of CAL27 cells, determined by cell counting at different time points (Fig. 8b; Additional file 1: Figure S6a). To better understand the regulation of CAL27 cell proliferation by circPVT1, we examined the cell cycle profile. As shown in Fig. 8c and in Additional file 1: Figure S6b, down-regulation of circPVT1 expression led to a significant decrease in the cell population in S phase and a significant increase in the cell population in G2 phase. An inhibitory effect on proliferation after circPVT1 down-regulation was also observed in colony-forming assay (Fig. 8d; Additional file 1: Figure S6c). Interestingly, circPVT1 down-regulation did not affect the migration of CAL27 cells (Fig. 8e; Additional file 1: Figure S6d). To better investigate the specific effect of circPVT1 in determining the phenotype of CAL27 cells, we reduced the level of its host gene PVT1 using a siRNA specific for the long noncoding RNA. siRNA-PVT1 decreased PVT1 expression by about 65% (Additional file 1: Figure S6e) at 48 h after transfection, but it did not affect the circPVT1 expression level (Additional file 1: Figure S6f). The siRNA-PVT1 effect was compared to the siRNAct control used above. Down-regulation of PVT1 did not impact on the proliferation rate of CAL27 cells, determined by cell counting and cell cycle analysis (Additional file 1: Figure S6g, h). Moreover, we did not obtain any statistically significant difference between siRNA-PVT1 and siRNAct with regard to the ability of CAL27 cells to form colonies (Additional file 1: Figure S6i). Finally, we evaluated the CAL27 cell line behavior in a migration assay upon down-regulation of PVT1. No specific role of PVT1 was observed in the migration of CAL27 cells (Additional file 1: Figure S6j).

The reduction of circPVT1 expression using both siRNA-circPVT1 and siRNAb-circPVT1 was also performed in Detroit 562 cells (Fig. 8a). The two siRNAs decreased circPVT1 expression by about 70 and 90%, respectively (Fig. 8a), compared to siRNAct. As seen in CAL27, the down-regulation of circPVT1 in Detroit 562 cells caused a significant reduction in their ability to form colonies compared to the control (Fig. 8f).

The phenotype following the down-regulation of circPVT1 was also observed in a human pharynx squamous cell carcinoma cell line (FaDu). The FaDu cell line has one missense mutation in codon 248 of exon 7 (mutp53R248L) and an additional heterozygous splicing site mutation of intron 6 with a frameshift and a premature stop codon, resulting in a truncated variant of p53. siRNA-circPVT1 and siRNAb-circPVT1 decreased circPVT1 expression in FaDu cells by about 70 and 90%, respectively, compared to siRNAct (Additional file 1: Figure S6k). After the down-regulation of circPVT1 in FaDu cells, their ability to form colonies was significantly reduced in comparison to the control (Additional file 1: Figure S6l).

Since cisplatin (CDDP) is one of the standard treatments for HNSCC patients, we assessed the potential positive effect of the down-regulation of circPVT1 expression on cisplatin-induced killing effects, using Detroit 562 cells. We found that circPVT1 down-regulation rendered Detroit 562 cells more prone to CDDP-induced killing effects (Fig. 8g, h). Both the half maximum effective concentration (EC50) and the half lethal concentration (LC50) were significantly reduced (Fig. 8i).

Finally, we studied the effect of circPVT1 over-expression on the CAL27 cell phenotype using the two vectors pcDNA3-circPVT1-a and pcDNA3-circPVT1-b (Additional file 1: Figure S7). circPVT1 over-expression with both vectors enhanced the proliferation rate of the CAL27 cells, determined by cell counting at different time points (Additional file 1: Figure S7a). Particularly, the vector pcDNA3-circPVT1-a had a stronger effect on CAL27 proliferation as confirmed by cell cycle analysis (Additional file 1: Figure S7a, b). It is also worth mentioning that circPVT1 over-expression by both vectors enhanced the capacity of CAL27 cells to form colonies (Additional file 1: Figure S7c).

### mut-p53 down-regulation reduces the proliferation of HNSCC cell lines

To investigate in more detail the relationship between mut-p53 and circPVT1, we evaluated whether the CAL27 phenotype after mut-p53 down-regulation followed the phenotype observed after circPVT1 down-regulation. Down-regulation of p53 significantly decreased the proliferation rate of CAL27 cells, determined by cell counting at different time points (Additional file 1: Figure S8a). To elucidate the details of the cell proliferation reduction, we performed cell cycle analysis, observing a significant increase in the cell population in G1 phase and a decrease in the cell population in S and G2 phases (Additional file 1: Figure S8b). These results indicate that mut-p53 down-regulation suppresses cell growth, promoting the block in G1 phase of the cell cycle. As for circPVT1, we observed a reduction in the colony number in a colony-forming assay (Additional file 1: Figure S8c). In contrast to circPVT1, mut-p53 protein was also involved in migration. In Additional file 1: Figure S8d we show a reduction of cell migration after p53 down-regulation of about 30% compared to the control. Finally, we performed an experiment to test the possible additive effect on CAL27 phenotype after concomitant down-regulation of p53 and circPVT1. We observed an additive effect on the proliferation rate as determined by cell counting (Additional file 1: Figure S8e). No other additive effect was evidenced from the cell cycle analysis or colony assay compared to the single down-regulation of p53 and circPVT1 (Additional file 1: Figure S8f, g).

### circPVT1 up-regulation pairs with the down-regulation of mut-p53-associated miR-497-5p

Among other putative circRNA functions, the current literature demonstrates that circRNAs have the potential to regulate miRNA expression [6, 15, 19]. Since circPVT1 expression was up-regulated in HNSCC, we focused our attention on the miRNAs down-regulated in our previous work [40].

**Fig. 9:**
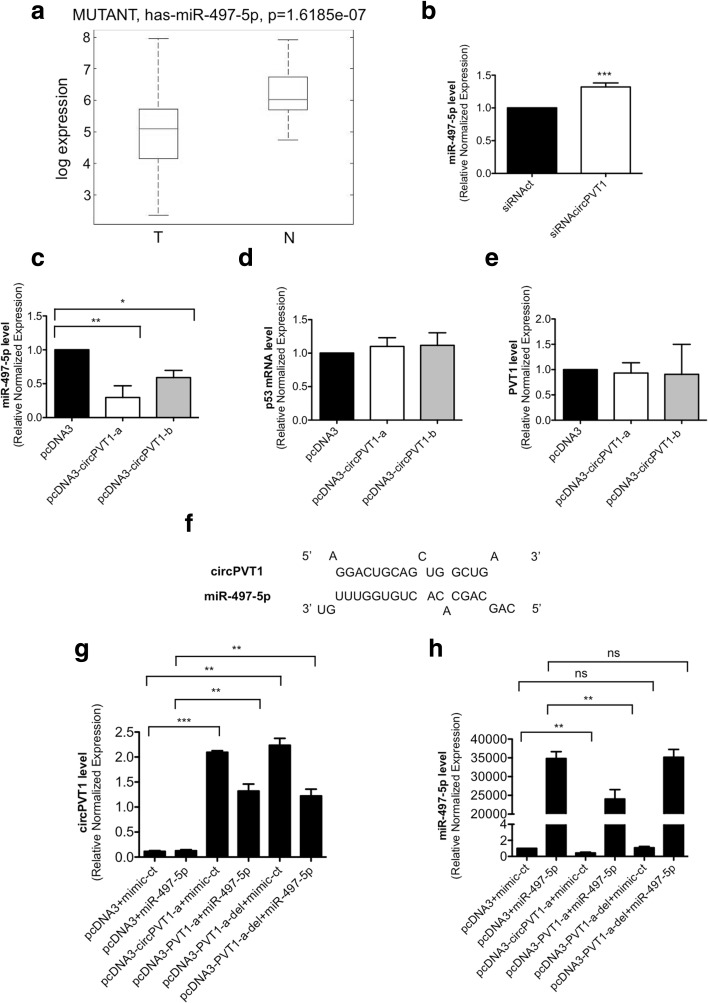
miR-497-5p expression in HNSCC patients and its regulation by circPVT1. **a** miRNA expression analysis as in [40] was used to evaluate miRNA-497-5p expression in tumoral (*T*) and non-tumoral (*N*) samples. **b** miRNA-497-5p level was detected by RT-qPCR in siRNAcircPVT1 and siRNAct transfected cells. **c** miRNA-497-5p level was detected by RT-qPCR in pcDNA3-circPVT1-a, pcDNA3-circPVT1-b, and pcDNA3 transfected cells. **d** p53 mRNA level was detected by RT-qPCR in pcDNA3-circPVT1-a, pcDNA3-circPVT1-b, and pcDNA3 transfected cells. **e** PVT1 level was detected by RT-qPCR in pcDNA3-circPVT1-a, pcDNA3-circPVT1-b, and pcDNA3 transfected cells. **f** circPVT1 binding site on miR-497-5p. **g** circPVT1 level was detected by RT-qPCR after the transfection of the indicated vectors and mimics. **h** miR-497-5p level was detected by RT-qPCR after the transfection of the indicated vectors and mimics. Data are shown as mean of three replicates ± standard deviation (Student’s test; *p < 0.05; **p < 0.01; ***p < 0.001; *ns* not significant)

In that study, we carried out miRNA expression profiling on 121 HNSCC samples and 66 non-tumoral counterparts, obtaining 49 miRNAs significantly associated with p53 status [40]. In particular, we found 44 miRNAs up-regulated and five miRNAs down-regulated. First of all, we evaluated the expression levels of the five miRNAs that were down-regulated, miR-497-5p, miR-99-5p, miR-370, miR-139-3p, and miR-1224-5p, in our patients bearing mut-p53, comparing tumor against non-tumoral tissues. We found a significant down-regulation of miR-497-5p (Fig. 9a), miR-99-5p, miR-370, miR-139-3p, and miR-1224-5p (Additional file 1: Figure S9a–d).

Secondly, we measured the correlation between circPVT1 and these miRNAs by Spearman and Pearson’s correlation (Additional file 2: Table S4) using the data on circPVT1 expression obtained from RT-qPCR as shown above, and the data on the five down-regulated miRNAs obtained from the miRNA expression profiling [40]. In detail, we analyzed 68 tumor samples with p53 mutations against 37 non-tumoral matched counterparts. We observed a significant negative relationship between circPVT1 and two of the miRNA selected: miR-99-5p and miR-497-5p (Additional file 2: Table S4). We measured the miRNA level in CAL27 cells after circPVT1 down-regulation using siRNAcircPVT1. In our in vitro model miR-99-5p was down-regulated after circPVT1 down-regulation (data not shown); in contrast, miR-139-3p, miR-370, and miR-1224-5p were undetectable by RT-qPCR due to their low expression levels. miR-497-5p was detectable by RT-qPCR and it was up-regulated after circPVT1 down-regulation, as expected from the data from patient analysis (Fig. 9b).

### circPVT1 binds to and regulates miR-497-5p expression

To investigate the effect of circPVT1 activity on miR-497-5p expression, we used the two vectors expressing circPVT1, pcDNA3-circPVT1-a and pcDNA3-circPVT1-b. Upon circPVT1 overexpression we evaluated the miR-497-5p level by RT-qPCR. miR-497-5p expression decreased by about 70% after the transfection of pcDNA3-circPVT1-a, and by about 50% after the transfection of pcDNA3-circPVT1-b (Fig. 9c). To verify if the activity of circPVT1 on miRNA-497-5p was specific, we evaluated the expression of the other two molecules possibly involved in the circPVT1 mechanism of action, i.e., mut-p53 and PVT1. After pcDNA3-circPVT1-a or -b transfection, we did not observe any modification in mut-p53 or PVT1 expression levels (Fig. 9d, e). To confirm that the circPVT1 was a direct regulator of miR-497-5p, we searched for the miR-497-5p binding site on the circPVT1 sequence (Fig. 9f). Then, we deleted the miR-497-5p binding site in the pcDNA3-circPVT1-a vector, generating the vector pcDNA3-circPVT1-a-del. The pcDNA3-circPVT1-a-del and pcDNA3-circPVT1-a vectors induced circPVT1 expression to similar levels (Fig. 9g), and after the co-transfection with miR-497-5p, the circPVT1 expression was still comparable between the two vectors (Fig. 9g). miR-497-5p expression was reduced after the transfection of pcDNA3-circPVT1-a alone or in combination with miR-497-5p (Fig. 9h). We did not observe any change in miR-497-5p expression after the transfection of pcDNA3-circPVT1-a-del with or without miR-497-5p (Fig. 9h). These data show that circPVT1 affected miR-497-5p expression through the selected binding site, which was specific and required for the miR-497-5p regulation. Finally, the regulation of miR-497-5p by circPVT1 might explain the presence of circPVT1 in the cytoplasm as well as the nucleus.

### miR-497-5p up-regulation in CAL27 cells mimics the phenotype induced by circPVT1 down-regulation

**Fig. 10:**
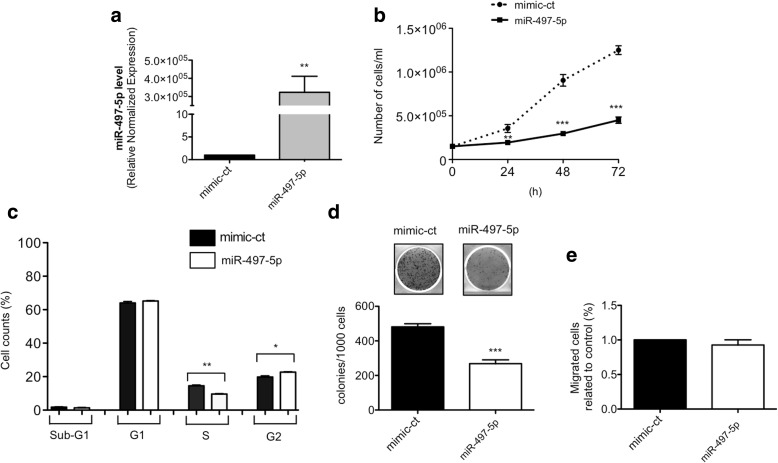
CAL27 phenotype after miRNA-497-5p over-expression. **a** miRNA-497-5p level was detected by RT-qPCR in miR-497-5p and in mimic-ct transfected cells. **b** Cell proliferation was determined by cell counting 24, 48, and 72 h after transfection with miR-497-5p and mimic-ct. **c** Propidium iodide flow cytometric assay was performed to analyze the cell cycle in miR-497-5p and mimic-ct transfected cells. **d** Colony formation assay was performed in miR-497-5p and mimic-ct transfected cells. *Top*: representative colony-forming assay. *Bottom*: quantification of three independent experiments by colony counting. **e** Migration assay was performed in miR-497-5p and mimic-ct transfected cells. Data are shown as mean of three replicates ± standard deviation (Student’s test; *p < 0.05; **p < 0.01; ***p < 0.001)

In order to confirm the relationship between circPVT1 and miR-497-5p, we evaluated if the CAL27 phenotype after miRNA up-regulation is the same as that observed after circPVT1 down-regulation. Up-regulation of miR-497-5p by a specific miRNA mimic at 48 h after transfection (Fig. 10a) significantly decreased the proliferation rate of CAL27 cells, determined by cell counting at different time points (Fig. 10b). By cell cycle analysis, we observed a significant decrease in the cell population in S phase and a significant increase in cell population in G2 phase (Fig. 10c). As observed for circPVT1, colony numbers were reduced in the colony-forming assay after miR-497-5p up-regulation (Fig. 10d) but with no effect on migration (Fig. 10e). Similar to the approach adopted to test possible additive effects of circPVT1 and p53 on the CAL27 phenotype, we performed simultaneous circPVT1 down-regulation and miR-497-5p up-regulation. No additive effect was observed on the CAL27 phenotype as shown in Additional file 1: Figure S10a–c. However, CAL27 proliferation and colony forming capacity were still affected by the simultaneous modulation of circPVT1 and miR-497-5p compared to the control.

### Aurka, mki67, and bub1 are genes involved in circPVT1 downstream oncogenic effects

**Fig. 11:**
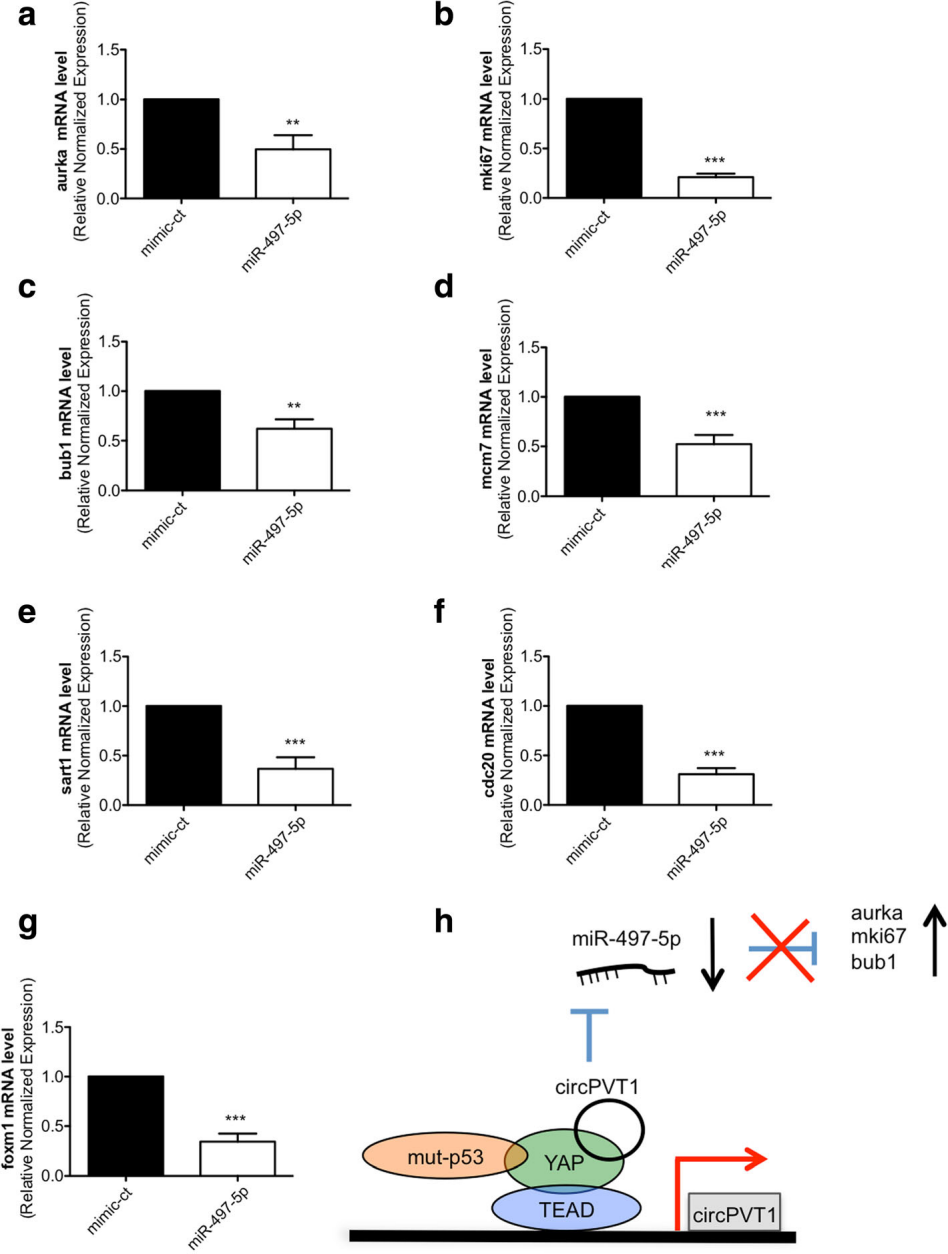
Expression analysis of selected genes after miRNA-497-5p over-expression and proposed model. **a** aurka, **b** mki67, **c** bub1, **d** mcm7, **e** sart1, **f** cdc20, and **g** foxm1 mRNA levels were detected by RT-qPCR in miR-497-5p and mimic-ct transfected cells. **h** Model of the oncogenic role of circPVT1 in tumor cells. Data are shown as mean of three replicates ± standard deviation (Student’s test; *p < 0.05;**p < 0.01; ***p < 0.001)

To investigate the molecular pathways involved in the CAL27 change of phenotype after miRNA-497-5p or circPVT1 modulation, we selected putative targets of miRNA-497-5p. Firstly, we selected genes based on negative correlation between miRNA-497-5p expression [40] and mRNA expression (data not shown), for a subgroup of HNSCC samples. Then, we focused on genes predicted as miRNA-497-5p targets and involved in cell proliferation. In particular, we selected aurka, mki67, bub1, mcm7, sart1, cdc20, and foxm1 and measured their expression at the transcriptional level after miR-497-5p over-expression (Fig. 11a–g). All genes selected were down-regulated after transfection with mimic-497-5p (Fig. 11a–g). Next, we measured the expression level of the selected genes after circPVT1 over-expression (Additional file 1: Figure S11). We obtained a significant overexpression of three genes: aurka, mki67, and bub1 (Additional file 1: Figure S11a-c), but not of the other genes (Additional file 1: Figure S11d-g). Since the expression of aurka, mki67, and bub1 was altered by both miR-497-5p and circPVT1 modulation, we propose that these genes were involved in the oncogenic role of circPVT1 in HNSCC. In line with this, a colony-forming assay upon down-regulation of bub1 expression, using three different siRNAs in CAL27 cells, revealed a reduced number of colonies compared to cells transfected with control siRNAs (Additional file 1: Figure S12a, b). This suggests that bub1 is among the downstream effectors of circPVT1 oncogenic activity in HNSCC.

## Discussion

CircRNAs have recently re-emerged as a class of endogenous RNAs with different roles in eukaryotic cells [5]. Of particular interest is the emerging oncogenic function of circRNAs, which might make them candidates for new biomarkers and therapeutic targets in cancer. Here, we investigated the role of circPVT1, a member of the circRNA family, in the pathogenesis of HNSCC.

We first investigated circPVT1 expression using the HNSCC cancer data set provided by TCGA. circPVT1 was found to be up-regulated in tumors hosting mut-p53 in comparison to unmatched non-tumoral samples. Similar findings were also found using a well-characterized collection of 115 HNSCC samples in which each tumor sample was compared to its matched non-tumoral tissue, minimizing inter-individual variation [40]. Indeed, circPVT1 expression determined by RT-qPCR was significantly up-regulated in tumor samples and in particular in those carrying TP53 mutations. This was not evidenced when circPVT1 expression was associated with FAT1 and CDKNA2 gene mutations in HNSCC, thereby mirroring circPVT1 as a non-coding mediator of mutant p53 oncogenic activities. Indeed, linear regression analysis showed a significant correlation between circPVT1 and mut-p53 in tumoral samples.

It is known that TP53 is the most frequent mutated gene in human cancers [46]. Moreover, p53 missense mutations not only determine the loss of its tumor-suppressive functions, but can also generate new proteins with oncogenic activities [47–50]. TP53 mutations are associated with decreased survival rate and increased risk of locoregional recurrence in HNSCC [40]. Using both the HNSCC cancer data set and our collection of HNSCC samples, we showed that circPVT1 also impacts on the survival of these patients. In particular, high circPVT1 levels are associated with a poorer overall survival in association with TP53 mutations. Moreover, circPVT1 plays a role in the resistance to cisplatin of HNSCC cell lines, but only in those carrying mutant p53 proteins. This further supports the role of circPVT1 in mut-p53-dependent pathways.

Our results show that circPVT1 is regulated by mut-p53 independent of its host gene PVT1. This mechanism appears to be unidirectional; in fact the down-regulation of circPVT1 did not influence mut-p53 protein expression. The analysis of the phenotype of HNSCC cell lines after down-regulation of either mut-p53 or circPVT1 expression revealed similar effects. We obtained in both cases a reduction of the malignant phenotype, showing that the two molecules work within the same molecular pathway. The role of circPVT1 as an oncogene was assessed also by circPVT1 overexpression. Cells over-expressing circPVT1 showed an increased capacity to proliferate and form colonies, further supporting the importance of circPVT1 in determining the oncogenic phenotype.

In an attempt to explain the link between mut-p53 and circPVT1 in tumors, we have established that YAP is a main regulator of circPVT1 expression at the transcriptional and post-transcriptional levels. Our data show that mut-p53 and TEAD are recruited at the same site where YAP binds the circPVT1 promoter, confirming the capacity of YAP and mut-p53 to interact with each other at promoter sites, and the important role of TEAD family transcription factors in mediating YAP-dependent gene expression [35, 43]. In fact, performing a DRB-4sU assay, we observed a decrease of nascent circPVT1 as a consequence of mut-p53, YAP, and TEAD1 down-regulation. YAP regulates circPVT1 also at the post-transcriptional level by binding the mature form of circular RNA, and mut-p53 acts as the stabilizer of the YAP/circPVT1 complex. In support of this model, we observed that when the mut-p53 protein was depleted, circPVT1 expression levels were reduced and the YAP/circPVT1 complex was lost.

Of note, we showed that circPVT1 uses its own promoter and not the promoter of its host gene. By ChIP assay experiments for p-Pol II, we showed that the newly identified circPVT1 promoter is a region that is transcriptionally active. Even if we did not exclude that the mut-p53/YAP/TEAD complex is also recruited at the PVT1 promoter level, our data support a mechanism according to which the mut-p53/YAP/TEAD complex works preferentially at the circPVT1 promoter level in the HNSCC context and in strict relation with mut-p53. It is reasonable to conclude that this status is achieved through the capacity of circPVT1 to regulate its own expression and to recruit preferentially the mut-p53/YAP/TEAD complex at its promoter region. In fact, we showed that the presence of circPVT1 in the nucleus is characterized by circPVT1 binding to the nuclear co-factor YAP. Moreover, by DRB-4sU and ChIP assay in circPVT1 over-expression conditions, we found, respectively, an increase in nascent circPVT1 production and an enrichment of Pol II and YAP recruitment at the circPVT1 promoter level.

We found that circPVT1 acts as an oncogene repressing the function of miRNA-497-5p, a miRNA associated with mut-p53 and that has been reported to have a tumor suppressor role in several cancers [51–54]. Here, we showed that miR-497-5p up-regulation significantly decreased the proliferation rate of CAL27 cells, which is consistent with its reported tumor suppressor function. Moreover, we originally demonstrated that circPVT1 is a direct regulator of miR-497-5p, impairing its tumor suppressor activity.

The circPVT1 downstream oncogenic effect included the up-regulation of aurka, mki67, and bub1, which are all putative targets of miRNA-497-5p and are involved in cell proliferation (Fig. 11h). We do not exclude that circPVT1 is also able to regulate other miRNAs. Indeed, we observed a significant negative relationship between circPVT1 and miR-99-5p (Additional file 2: Table S4), even if this relationship was not confirmed in our in vitro model. The study of other miRNAs regulated by circPVT1 will be the subject of future work.

Collectively, these findings depict a network in which gain of function mutant p53 proteins trigger the activation of a downstream non-coding effector, demonstrated here by the binding of circPVT1 and miR-497-5p, which leads to unrestrained proliferation through the aberrant enhancement of the expression of cell cycle regulated genes. The involvement of YAP and TEAD as critical components of the Hippo tumor suppressor pathway in the context of mutant p53 activity might represent an additional proof of the aberrant crosstalk between two distinct tumor suppressor pathways. Indeed, TP53 mutations lead to the production of mutant p53 proteins that engage physically with YAP and TEAD and might subvert the tumor suppressor into oncogenic activities.

## Conclusions

We found that circPVT1 behaves as an oncogene in HNSCC and that the mut-p53/YAP/TEAD complex transcriptionally regulates its expression. Although further studies are necessary to thoroughly elucidate the molecular pathway in which circPVT1 is involved, these findings significantly advance our understanding of the circRNAs’ mechanism of action. Further to providing new insights into the biological function of circRNAs in cancer, our data might contribute to identify new candidate non-coding biomarkers such as circRNAs specifically associated with TP53 mutations in HNSCC. This might be useful for diagnostic and therapeutic strategies in HNSCC.

## Methods

### Study population and clinical samples

The study population and clinical samples are described in [40]. Briefly, the study population includes 115 prospectively enrolled patients with histologically confirmed primary HNSCC undergoing curative treatment at the Otolaryngology Head and Neck Surgery Department. Only patients who did not receive any anticancer therapy before surgery were included in the study. Only HNSCC patients who developed local recurrence 1 month after surgery and with a follow-up ≥ 12 months were considered for the prognostic study. Two biopsies, from tumor and histologically normal tissue, were collected at surgery and preserved in RNA later (Ambion, Austin, TX, USA) from each patient. The histologically normal tissue was taken in correspondence with surgical resection margins and are described in the text as non-tumoral tissue.

### P53 mutational analysis

The p53 mutational analysis is described in [40].

### Cells

CAL27, FaDu, A253, and H1299 cell lines were maintained in RPMI-1640 medium (Invitrogen); the Detroit 562 cell line was maintained in DMEM (Invitrogen). The medium was supplemented with 10% fetal bovine serum (FBS; Invitrogen), penicillin (100 U/ml; GIBCO), and streptomycin (200 mg/ml; GIBCO) at 37 °C in a 5% CO_2_ atmosphere.

### Quantification of circPVT1 molecules per cell

We used the standard curve method to obtain the nanograms (ng) of circPVT1. First, we found the concentration for each sample on the standard curve; then we multiplied the concentration by the dilution factor for each sample. We obtained the nanogram amount of circPVT1 in the two fractions: 0.82 ng in the nucleus and 4.48 ng in the cytosol for Detroit cells; 0.83 ng in the nucleus and 4.22 ng in the cytosol for CAL27 cells. Next, we applied the following formula to get the number of copies (molecules) of our PCR product:

$$ \frac{\left( circPVT1\  ng\ast 6.022x{10}^{23} molecules/ mole\right)}{\left( circPVT1\  length\ \left(410\  bp\right)\ast 1x{10}^9 ng/g\ast 660\ g/ mole\right)}. $$

Finally, we divided the above result by the number of cells (5.88 × 10^6^ for Detroit cells and 4.35 × 10^6^ for CAL27 cells).

### RNAse R treatment

The RNAse treatment was performed using Ribonuclease R (RNase R) according to the manufacturer’s recommendations (Epicentre). In particular, 2 μg of RNA were treated with 10 U of RNase R at 37 °C for 30 minutes in 10× Reaction Buffer. The untreated samples were incubated at the same conditions in 10× Reaction Buffer without the RNase R.

### Cell transfection

The transfections of siRNAs and mimicRNAs were performed using Lipofectamine RNAiMax (Life Technologies). The transfections of vectors were performed using Lipofectamine 2000 (Life Technologies). All experiments were conducted according to the manufacturer’s recommendations. For mature miR-497-5p expression, we used the mirVana miR-495-5p mimic (Ambion) at a final concentration of 5 nM and as control we used the mirVana miRNA mimic, Negative Control #1 (Ambion), at the same concentration. The transfection of vectors and mimicRNAs in the same experiment were performed using Lipofectamine RNAiMax (Life Technologies) for mimicRNAs and, 24 h after the first transfection, a second transfection for the vectors was performed using Lipofectamine 2000 (Life Technologies). The mut-p53 overexpression in A253 cells was performed using 5 μg pcDNA3-p53-R175H [55], and the same amount of pcDNA3 was used as control. For the H1299 RIP assay we used 5 μg pcDNA3-p53-R175H + 8 μg pcDNA3- circPVT1-a and 13 μg pcDNA3 vector as control. For the CAL27 RIP assay of overexpression, we used 5 μg YAP-Flag [56] + 8 μg pcDNA3-circPVT1-a and 13 μg pcDNA3 vector as control. For the CAL27 RIP assay in silencing conditions we used 320 pmol siRNAct, 320 pmol siRNA-YAP, and 320 pmol siRNAp53. The circPVT1 overexpression in CAL27 cells was performed using 4 μg pcDNA3-circPVT1-a or -b and 4 μg pcDNA3 vector as control. siRNAp53 smart pool was used at 80 pmol final concentration. Custom siRNAs were used at the following final concentrations: siRNAct, 150 pmol; siRNAct1, 150 pmol; siRNAct2, 150 pmol; siRNAbct1, 150 pmol; siRNAbct2, 150 pmol; siRNAcircPVT1, 150 pmol (siRNA overlaps the splice junction); siRNAb-circPVT1, 150 pmol (siRNA overlaps the splice junction); siRNA-PVT1, 300 pmol; siRNAp53, 150 pmol; siRNAp53 3′ UTR, 80 pmol; siRNA-YAP, 160 pmol; siRNA-YAP2, 80 pmol; siRNA-TAZ, 80 pmol siRNA-TAZ-1 + 80 pmol siRNA-TAZ-2; siRNA-TEAD, 40 pmol siTEAD1/3/4 + 40 pmol siTEAD2 + 40 pmol siTEAD1 ex 5 + 40 pmol siTEAD1 ex 8; siRNA-BUB1_1, 160 pmol; siRNA-BUB1_2, 160 pmol; siRNA-BUB1_3, 160 pmol.

### Construction of circPVT1 expression vectors

pcDNA3-circPVT1-a was constructed by PCR amplification of the circPVT1 locus, including 954 bp upstream and 53 bp downstream of the nonlinear splice sites. We used the primers F1 and R1 designed to incorporate HindIII and NotI restriction sites and 6 bp of extra random sequence to aid in restriction digestion. The amplified region was cloned into the HindIII/NotI sites of pcDNA3 (Invitrogen). For construction of pcDNA3-circPVT1-b, an 895 bp DNA stretch upstream of the splice acceptor was amplified and inserted downstream in the reverse orientation in an XhoI/ApaI digested pcDNA3-circPVT1-a, using the primers F2 and R2 designed to incorporate ApaI and XhoI restriction sites and 6 bp of extra random sequence to aid in restriction digestion.

The pcDNA3-circPVT1-a-del was generated utilizing the pcDNA3-circPVT1-a plasmid as template. We deleted the miR-497-5p binding site using the QuikChange II XL site-directed mutagenesis kit (Agilent Technologies). Primer sequences are listed in Additional file 3: Table S5 and designated circPVT1_delmiR-497-5p F and circPVT1_delmiR-497-5p R. The miR-497-5p binding site was identified by RNAhybrid [57].

### Cell proliferation assay

Cell proliferation was determined by viable cell counting. We seeded 1.5 × 10^5^ cells in six-well plates in duplicate and grown for 72 h. Cell counting was performed after 24, 48, and 72 h mixing an aliquot of cells 1:1 with Trypan Blue dye (Invitrogen).

### Cell cycle analysis

We fixed 10^6^ cells in cold ethanol and these were then washed, resuspended in phosphate-buffered saline containing 50 μg/ml propidium iodide (PI; Sigma-Aldrich) and 50 μg/ml RNAase A, and analyzed by flow cytometry on a Millipore Guava® easyCyte 8HT.

### Colony-forming assay

We plated 10^3^ cells in 60 mm dishes and incubated them at 37 °C in a 5% CO_2_ atmosphere for colony formation. After 10 days, colonies were stained with crystal violet (Sigma-Aldrich) for 30 min and counted.

### Viability assays

Cell viability was assessed using an ATPlite assay (Perkin Elmer, Massachusetts, USA) accordingly to the manufacturer’s instructions. We seeded 800 cells in 96-well plates in 200 μl of media. Silencing was performed in reverse transfection and, after 24 h, cells were treated as indicated with cisplatin (CDDP) for a further 72 h. Each plate was evaluated immediately on a microplate reader (Expire Technology, Perkin Elmer). Each sample was assayed in triplicate.

### Transwell migration assays

Transfected cells were detached and counted. A migration assay was performed using a 24-well plate. We seeded 6 × 10^4^ cells, in a volume of 500 μl RPMI-1640 without FBS, in the upper chamber with an 8-mm pore size filter (BD Falcon, Franklin Lakes, NJ, USA) while the bottom chamber of the transwell was filled with 700 μl of RPMI-1640 with 10% FBS. Cells were allowed to migrate for 36 h in a humidified incubator at 37 °C and 5% CO_2_. Migrated cells, which had attached to the outside of the filter, were visualized by staining with DAPI (Sigma-Aldrich) and counted under a Zeiss LSM 510 laser scanning fluorescence confocal microscope.

### Western blot analysis

Cells were homogenized on ice for 30 min in a lysis buffer composed by 50 mM, Hepes pH 7.5, 5 mM EDTA pH 8.0, 10 mM MgCl_2_, 150 mM NaCl, 50 mM NaF, 20 mM β-glicerophosphate, 0.5% NP40, 0.1 mM sodium orthovanadate, 1 mM PMSF, 1 mM dithiothreitol (DTT), and protease inhibitor cocktail (Roche). Lysates were clarified by centrifugation for 10 min, max speed, at 4 °C. Proteins (30 μg/lane) were separated on 10% SDS-polyacrylamide gels and transferred to nitrocellulose membranes. Immunoblots were probed with the following primary antibodies: mouse monoclonal anti-p53 (DO1; Oncogene Science Uniondale, NY, USA), rabbit polyclonal anti-YAP (Santa Cruz Biotechnology), and mouse monoclonal anti-GAPDH (Calbiochem). Immunostained bands were detected by a chemiluminescent method.

### RNA extraction, reverse transcription, and RT-qPCR

The total RNA was extracted with TRizol (Thermo Fisher Scientific) following the manufacturer’s instructions and quantified using a Nanodrop (Thermo Scientific). The concentration, purity, and quality of total RNA were assessed using a Nanodrop TM 1000 spectrophotometer (Nanodrop Technologies). Reverse Transcription of miRNAs and RT-qPCR quantification of miRNA expression were performed by TaqMan MicroRNA RT assay and TaqMan MiRNA® Assay (Thermo Fisher Scientific), respectively, according to the manufacturer’s protocol. RNU44 and RNU48 were used as endogenous controls to standardize miRNA expression. For circPVT1, PVT1, and gene expression analysis, reverse transcription and RT-qPCR were performed using MMLV RT (Invitrogen) and SYBR Green® Assays (Applied Biosystems), respectively, according to the manufacturers’ instructions. For the detection of the circPVT1 expression level we used divergent primers that produce a PCR product only when the template is circular. circPVT1 expression in patients was normalized to the geometric mean of H3, GAPDH, and RPL19. In the other RT-qPCRs the expression of the genes was normalized to the GAPDH level.

### ChIP experiments

We performed 1% formaldehyde cross-linking and ChIP as described in [35]. The chromatin solution was immunoprecipitated with sheep polyclonal anti-p53 Ab7 antibody (Merck Millipore), rabbit polyclonal anti-YAP (H-125; Santa Cruz), rabbit polyclonal anti-Pol II (N-20; Santa Cruz), mouse monoclonal anti-TEF-1 (anti-TEAD-1, BD Transduction Laboratories), and ChIP-grade mouse monoclonal [4H8] anti-RNA polymerase II CTD repeat YSPTSPS (phospho S5; Abcam). The immunoprecipitations were performed using Pierce ChIP-grade Protein A/G magnetic beads (Thermo Fisher Scientific). The immunoprecipitated and purified chromatin was subjected to RT-qPCR. The promoter occupancy was analyzed by RT-qPCR using the SYBR Green assay (Applied Biosystems). Normalization was performed to the amount of input chromatin.

### RNA immunoprecipitation

Untreated CAL27 cells were detached and resuspended in freshly prepared nuclear isolation buffer, kept on ice for 5 min (with frequent mixing) and stored at −80 °C. The cell lysate was twice frozen and thawed before use. Lysate was passed through a 27.5 gauge needle four times to promote nuclear lysis and centrifuged at 14,000 × g for 10 min at 4 °C. An aliquot of lysate (10 μl) was used as input for western blot analysis. For immunoprecipitation, the lysates were incubated with 50 μl of Dynabeads protein G (Thermo Fisher Scientific) and 7 μl of anti-p53 Ab7 antibody (Merck Millipore), rabbit polyclonal anti-YAP (H-125; Santa Cruz Biotechnology) or sheep serum, used as control, under constant shaking at room temperature for 1 h. Dynabeads protein G were prepared according to the manufacturer’s instructions. Following the immunoprecipitation, 10% of the beads were used for weatern blot analysis and the other part was used to isolate co-precipitated RNAs by resuspending beads in TRIzol (Thermo Fisher Scientific) according to the manufacturer’s instructions.

### DRB-4sU assay

Transfection of CAL27 cells was performed as indicated above. Cells were incubated with 100 μM of 5,6-dichlorobenzimidazole 1-β-D-ribofuranoside (DRB; Sigma, D1916) for 3 h to block Pol II transcription. Transcription was recovered after DRB release and newly transcribed RNA was labeled with 350 μM of 4-thiouridine (4sU; Sigma, T4509) for 2 h. Cells were trypsinized and pelleted. TRIzol (Invitrogen) was added to stop transcription and total RNA was extracted. Total RNA (15 μg) was used for biotinylation and purification of 4sU-labeled nascent RNA, for each point. 4sU-labeled RNA was incubated in biotinylation buffer (10 mM HEPES pH 7.5, 1 mM EDTA) with 5 μg of MTSEA Biotin-XX (90066/9006-1 Biotium) for 1.5 h with rotation. MTSEA Biotin-XX was dissolved in dimethylformamide (DMF; Sigma, D4551) at a concentration of 10 mg/ml. Next, unbound Biotin-XX was removed by an equal volume of phenol:chloroform:isoamyl alcohol (15593-031, Invitrogen) and RNA was precipitated at 13,000 rpm for 20 min at 4 °C with 1:10 volume of 5 M NaCl and an equal volume of isopropanol. The RNA pellet was washed with 75% ethanol and resuspended in 50 μl nuclease-free H_2_O. 4sU-labeled and unlabeled RNA was separated by using 200 μl Dynabeads MyOne Streptavidin C1 (65001, Invitrogen) at room temperature for 20 min according to the manufacturer’s instructions. Beads were washed five times with 0.8 ml of washing buffer (100 mM Tris pH 7.4, 10 mM EDTA, 1 M NaCl, 0.1% Tween 20). Nascent RNA was eluted with 100 μl 0.1 M DTT twice and was precipitated with 40 μl of 4 M LiCl, 2 μl glycogen, and 600 μl ice-cold ethanol. The RNA pellet was resuspended in 20 μl nuclease free H_2_O and incubated at 65 °C for 10 min at 400 rpm. Reverse transcription and RT-qPCR quantification were performed as described above.

### circPVT1 and clinical features

A logistic regression model was fitted to evaluate association with clinical variables. Statistical significance was set at 5%.

Kaplan-Meier analysis was used for overall survival and the log-rank test was used to assess differences between curves with high and low circPVT1 intensity. We defined the group of patients with low circPVT1 expression as samples with expression lower than median(X)-σ/2, where X is the circPVT1 expression distribution and σ the standard deviation.

The EC50 and the LD50 were determined using the Compusyn Software [58].

### Analysis of TCGA HNSCC exon expression data

Normalized TCGA HNSCC exon expression analysis was performed using 31 non-tumoral and 263 tumor samples (we obtained information for 129 mut-p53 and 57 wt-p53 samples). The chromosomal intervals are related to the human genome GRch37/hg19. Deregulation of specific exons in different subgroups of samples was assessed by a two-sided Wilcoxon rank sum test.

### Analysis of PVT1 and circPVT1 promoters

We used the Promoter 2.0 prediction and the Lasagna 2.0 web-tool to analyze the PVT1 and circPVT1 promoters. The promoter sequences are related to the human genome GRch38/hg38.

### In silico prediction targets

In silico prediction of miRNA-497-5p targets was performed using miRwalk2 [59]. We selected targets predicted from three different software and chose according to the negative correlation between miRNA-497-5p expression [40] and mRNA expression analysis of 14 patients with TP53 mutations and nine normal matched patients.

### Quantification and statistical analysis

GraphPad Prism was used to determine the statistical significance of the in vitro experiments. MATLAB was used to determine the statistical significance of the data involving patient samples. For each figure, relevant information for assessing the accuracy and precision of the analysis is included in the accompanying legend.

## Additional files

Additional file 1: Supplementary results and figures. (DOCX 3840 kb)

Additional file 2: Table S1. Baseline characteristics of patient 78. **Table S2.** List of patients ordered on the basis of circPVT1 expression detected by RT-qPCR, and TP53 status. **Table S3.** Results of univariate linear regression analysis with clinical variables in tumoral samples. **Table S4.** Correlation analysis of circPVT1 and p53-associated miRNAs in HNSCC. (XLSX 58 kb)

Additional file 3: Table S5. Sequence based reagents. (XLSX 14 kb)
