# Supplementary material for: The oncogenic role of circPVT1 in head and neck squamous cell carcinoma is mediated through the mutant p53/YAP/TEAD transcription-competent complex
Source: Genome Biol. 2017 Dec 20;18:237. doi: 10.1186/s13059-017-1368-y (PMC5738800; doi:10.1186/s13059-017-1368-y)
Supplement: Supplementary file 1 — Supplementary results and figures. (DOCX 3840 kb) [file 13059_2017_1368_MOESM1_ESM.docx]

**Table of Contents**

Supplementary results 1: circPVT1 Sanger-sequencing results.........…….2

Supplementary Figures………………………………………………………....7

Figure S1……………………………………………………………………...….7

Figure S2………………………………………………………………………....8

Figure S3…………………………………………………………………………11

Figure S4…………………………………………………………………………13

Figure S5…………………………………………………………………………15

Figure S6…………………………………………………………………………17

Figure S7…………………………………………………………………………19

Figure S8…………………………………………………………………………21

Figure S9…………………………………………………………………………23

Figure S10………………………………………………………………………..24

Figure S11………………………………………………………………………..25

Figure S12………………………………………………………………………..27

**Supplementary results 1: circPVT1 Sanger-sequencing results**

We used the divergent primers P1 and P2 to perform the Sanger-sequencing in 20 patients:

**P2** **TACTTGAACGAAGCTCCATGCAGC**

**P1 CGACTCTTCCTGGTGAAGCATCTGAT**

We reported below the circPVT1 sequence with the primer P2 indicated in blue, the primer P1 in pink, and the two regions amplified by the sequencing indicated in turquoise and green:

GCCTGATCTTTTGGCCAGAAGGAGATTAAAAAGATGCCCCTCAAGATGGCTGTGCCTGTCAGCTGCATGGAGCTTCGTTCAAGTATTTTCTGAGCCTGATGGATTTACAGTGATCTTCAGTGGTCTGGGGAATAACGCTGGTGGAACCATGCACTGGAATGACACACGCCCGGCACATTTCAGGATACTAAAAGTGGTTTTAAGGGAGGCTGTGGCTGAATGCCTCATGGATTCTTACAGCTTGGATGTCCATGGGGGACGAAGGACTGCAGCTGGCTGAGAGGGTTGAGATCTCTGTTTACTTAGATCTCTGCCAACTTCCTTTGGGTCTCCCTATGGAATGTAAGACCCCGACTCTTCCTGGTGAAGCATCTGATGCACGTTCCATCCGGCGCTCAGCTGGGCTTGAG

Results for patients with mutant p53 (The identification numbers indicate the patients. T; Tumor):

**7T**

P2

CCTTAAGTCGGGCGCTAGCTGGGCTTGAGGCCTGATCTTTTGGCCAGAAGGAGATTAAAAAGATGCCCCTCAAGATGGCTGTGCCTGTCAGCTGCATGGAGCTTCGTTCAAGTAA

P1 (Reverse strand)

GGTGTCAGTCGTCGTTGAGGGGCATAGTTTTTATCTCCTTCTGGCCAAAAGATCAGGCCTCAAGCCCAGCTGAGCGCCGGATGGAACGTGCATCAGATGCTTCACCAGGAAGAGTCGA

**17T**

P2

GCATAATCGGGCGCTAGCTGTAACTAGAGGCCTGATCTTTTGGCCAGAAGGAGATTAAAAAGATGCCCCTCAAGATGGCTGTGCCTGTCAGCTGCATGGAGCTTCGTTCAAGTAA

P1 (Reverse strand)

TTAGTCAGTCAATCTTGAGGGGCATCTTTTTATCTCCTTCTGGCCAAAAGATCAGGCCTCAAGCCCAGCTGAGCGCCGGATGGAACGTGCATCAGATGCTTCACCAGGAAGAGTCGA

**29T**

P2 CACATCGTCCAGCGCTAGCTGGGCTTGAGGCCTGATCTTTTGGCCAGAAGGAGATTAAAAAGATGCCCCTCAAGATGGCTGTGCCTGTCAGCTGCATGGAGCTTCGTTCAAGTAA

P1 (Reverse strand)

GTGACGTCATCTTGAGGGGCATCTTTTTATCTCCTTCTGGCCAAAAGATCAGGCCTCAAGCCCAGCTGAGCGCCGGATGGAACGTGCATCAGATGCTTCACCAGGAAGAGTCGA

**37T**

P2

CACTGTCACGCCAGCGTCTAGCGTACCTCCTGATAACCTGATCTTTTGGCCAGAAGGAGATTAAAAAGATGCCCCTCAAGATGGCTGTGCCTGTCAGCTGCATGGAGCTTCGTTCAAGTAA

P1 (Reverse strand)

GTAATAGTCAATCGTTGAGAGGCATAGTTTTTATCTCCTTCTGGCCAAAAGATCAGGCCTCAAGCCCAGCTGAGCGCCGGATGGAACGTGCATCAGATGCTTCACCAGGAAGAGTCGA

**49T**

P2 GAATAATCGGGCGCTAGCTGGGCTTGAGGCCTGATCTTTTGGCCAGAAGGAGATTAAAAAGATGCCCCTCAAGATGGCTGTGCCTGTCAGCTGCATGGAGCTTCGTTCAAGTAA

P1 (Reverse strand)

TTGAAGTCAATCTTGAGGGGCATCTTTTTATCTCCTTCTGGCCAAAAGATCAGGCCTCAAGCCCAGCTGAGCGCCGGATGGAACGTGCATCAGATGCTTCACCAGGAAGAGTCGA

**52T**

P2 CGTCATCCGGCGCTAGCTGGGCTTGAGGCCTGATCTTTTGGCCAGAAGGAGATTAAAAAGATGCCCCTCAAGATGGCTGTGCCTGTCAGCTGCATGGAGCTTCGTTCAAGTAA

P1 (Reverse strand)

GGAGAAGTCATCTTGAGGGGCATCTTTTTATCTCCTTCTGGCCAAAAGATCAGGCCTCAAGCCCAGCTGAGCGCCGGATGGAACGTGCATCAGATGCTTCACCAGGAAGAGTCGA

**55T**

P2 CCCCGTTAATCCGGCGCTAGCTGGGCTTGAGGCCTGATCTTTTGGCCAGAAGGAGATTAAAAAGATGCCCCTCAAGATGGCTGTGCCTGTCAGCTGCATGGAGCTTCGTTCAAGTAA

P1 (Reverse strand)

GAGACGTCATCTTGAGGGGCATCTTTTTATCTCCTTCTGGCCAAAAGATCAGGCCTCAAGCCCAGCTGAGCGCCGGATGGAACGTGCATCAGATGCTTCACCAGGAAGAGTCGA

**61T**

P2 CCCCGGCAGGCAAGCGACTAGCTGTCTAATTGAGGCCTGATCTTTTGGCCAGAAGGAGATTAAAAAGATGCCCCTCAAGATGGCTGTGCCTGTCAGCTGCATGGAGCTTCGTTCAAGTAA

P1 (Reverse strand)

GGGTCGGACATCTTGAGGGCATCTTTTTATCTCCTTCTGGCCAAAAGATCAGGCCTCAAGCCCAGCTGAGCGCCGGATGGAACGTGCATCAGATGCTTCACCAGGAAGAGTCGA

**84T**

P2 GGCCTCATCAGCGACTATCGTGTCAGTTGAGGCCTGATCTTTTGGCCAGAAGGAGATTAAAAAGATGCCCCTCAAGATGGCTGTGCCTGTCAGCTGCATGGAGCTTCGTTCAAGTAA

P1 (Reverse strand)

GGGGTAAGTCAGTCGTTGAGGGGCATAGTTTTTATCTCCTTCTGGCCAAAAGATCAGGCCTCAAGCCCAGCTGAGCGCCGGATGGAACGTGCATCAGATGCTTCACCAGGAAGAGTCGA

**116T**

P2 CAGTAATCGGCGCTAGCTGGGCTTGAGGCCTGATCTTTTGGCCAGAAGGAGATTAAAAAGATGCCCCTCAAGATGGCTGTGCCTGTCAGCTGCATGGAGCTTCGTTCAAGTAA

P1 (Reverse strand)

GTGGCAGTATCTTGAGGGGCATCTTTTTATCTCCTTCTGGCCAAAAGATCAGGCCTCAAGCCCAGCTGAGCGCCGGATGGAACGTGCATCAGATGCTTCACCAGGAAGAGTCGA

Results for patients with wild type p53 (The identification numbers indicate the patients. T; Tumor):

**1T**

P2 GAGCTCTTCTGCGGCGCTTAGCTGTCGCTTGAGGCCTGATCTTTTGGCCAGAAGGAGATTAAAAAGATGCCCCTCAAGATGGCTGTGCCTGTCAGCTGCATGGAGCTTCGTTCAAGTAA

P1 (Reverse strand)

CGTGGAAAGTCCATCTTGAGGGCATCTTTTTATCTCCTTCTGGCCAAAAGATCAGGCCTCAAGCCCAGCTGAGCGCCGGATGGAACGTGCATCAGATGCTTCACCAGGAAGAGTCGA

**2T**

P2 GAGTACGGCAATGCGACTATGTGTCGGTTGAGGCCTGTATCTTTTGGCCAGAAGGAGATTAAAAAGATGCCCCTCAAGATGGCTGTGCCTGTCAGCTGCATGGAGCTTCGTTCAAGTAA

P1 (Reverse strand)

GGTGGTCGTCCTTCGTTGAGGGCATAGTTTTTATCTCCTTCTGGCCAAAAGATCAGGCCTCAAGCCCAGCTGAGCGCCGGATGGAACGTGCATCAGATGCTTCACCAGGAAGAGTCGA

**42T**

P2 CAATAATCGGCGCTAGCTGGGCTTGAGGCCTGATCTTTTGGCCAGAAGGAGATTAAAAAGATGCCCCTCAAGATGGCTGTGCCTGTCAGCTGCATGGAGCTTCGTTCAAGTAA

P1 (Reverse strand)

GTGTAGTCATCTTGAGGGGCATCTTTTTATCTCCTTCTGGCCAAAAGATCAGGCCTCAAGCCCAGCTGAGCGCCGGATGGAACGTGCATCAGATGCTTCACCAGGAAGAGTCGA

**54T**

P2 CACTCATCGGCGCTAGCTGGGCTTGAGGCCTGATCTTTTGGCCAGAAGGAGATTAAAAAGATGCCCCTCAAGATGGCTGTGCCTGTCAGCTGCATGGAGCTTCGTTCAAGTAA

P1 (Reverse strand)

GGAGGACAGTCATCTTGAGGGGCATCTTTTTATCTCCTTCTGGCCAAAAGATCAGGCCTCAAGCCCAGCTGAGCGCCGGATGGAACGTGCATCAGATGCTTCACCAGGAAGAGTCGA

**77T**

P2 CACTAAGCCGGCGCTATCAGTAACTTGAGGCCTGATCTTTTGGCCAGAAGGAGATTAAAAAGATGCCCCTCAAGATGGCTGTGCCTGTCAGCTGCATGGAGCTTCGTTCAAGTAA

P1 (Reverse strand)

GGCAGACCATCGTTGAGGGGCATCTTTTTATCTCCTTCTGGCCAAAAGATCAGGCCTCAAGCCCAGCTGAGCGCCGGATGGAACGTGCATCAGATGCTTCACCAGGAAGAGTCGA

**79T**

P2 CATCTATCCGGCGCTAGCTGGGCTTGAGGCCTGATCTTTTGGCCAGAAGGAGATTAAAAAGATGCCCCTCAAGATGGCTGTGCCTGTCAGCTGCATGGAGCTTCGTTCAAGTAA

P1 (Reverse strand)

GAGTCAGTATCTTGAGGGGCATCTTTTTATCTCCTTCTGGCCAAAAGATCAGGCCTCAAGCCCAGCTGAGCGCCGGATGGAACGTGCATCAGATGCTTCACCAGGAAGAGTCGA

**89T**

P2 CAATTATCGGCGCTAGCTGGGCTTGAGGCCTGATCTTTTGGCCAGAAGGAGATTAAAAAGATGCCCCTCAAGATGGCTGTGCCTGTCAGCTGCATGGAGCTTCGTTCAAGTAA

P1 (Reverse strand)

GTGGCAGTCATCTTGAGGGGCATCTTTTTATCTCCTTCTGGCCAAAAGATCAGGCCTCAAGCCCAGCTGAGCGCCGGATGGAACGTGCATCAGATGCTTCACCAGGAAGAGTCGA

**90T**

P2 GGGATCATCCGGGCGCTAGCTGGGCTTGAGGCCTGATCTTTTGGCCAGAAGGAGATTAAAAAGATGCCCCTCAAGATGGCTGTGCCTGTCAGCTGCATGGAGCTTCGTTCAAGTAATGAATAAACCTAGCGACCTA

P1 (Reverse strand)

GGTGTCAGTCCATCTTGAGGGGCATCTTTTTATCTCCTTCTGGCCAAAAGATCAGGCCTCAAGCCCAGCTGAGCGCCGGATGGAACGTGCATCAGATGCTTCACCAGGAAGAGTCGA

**122T**

P2 CACTAATCAGCGACTATCTGTCAGTTGAGGCCTGATCTTTTGGCCAGAAGGAGATTAAAAAGATGCCCCTCAAGATGGCTGTGCCTGTCAGCTGCATGGAGCTTCGTTCAAGTAA

P1 (Reverse strand)

GTGGACGGTCGTCGTTGAGAGGCATAGTTTTTATCTCCTTCTGGCCAAAAGATCAGGCCTCAAGCCCAGCTGAGCGCCGGATGGAACGTGCATCAGATGCTTCACCAGGAAGAGTCGA

**126T**

P2 CCATTATCGGCGCTAGCTGGGCTTGAGGCCTGATCTTTTGGCCAGAAGGAGATTAAAAAGATGCCCCTCAAGATGGCTGTGCCTGTCAGCTGCATGGAGCTTCGTTCAAGTAA

P1 (Reverse strand)

GAGACAGTATCTTGAGGGGCATCTTTTTATCTCCTTCTGGCCAAAAGATCAGGCCTCAAGCCCAGCTGAGCGCCGGATGGAACGTGCATCAGATGCTTCACCAGGAAGAGTCGA


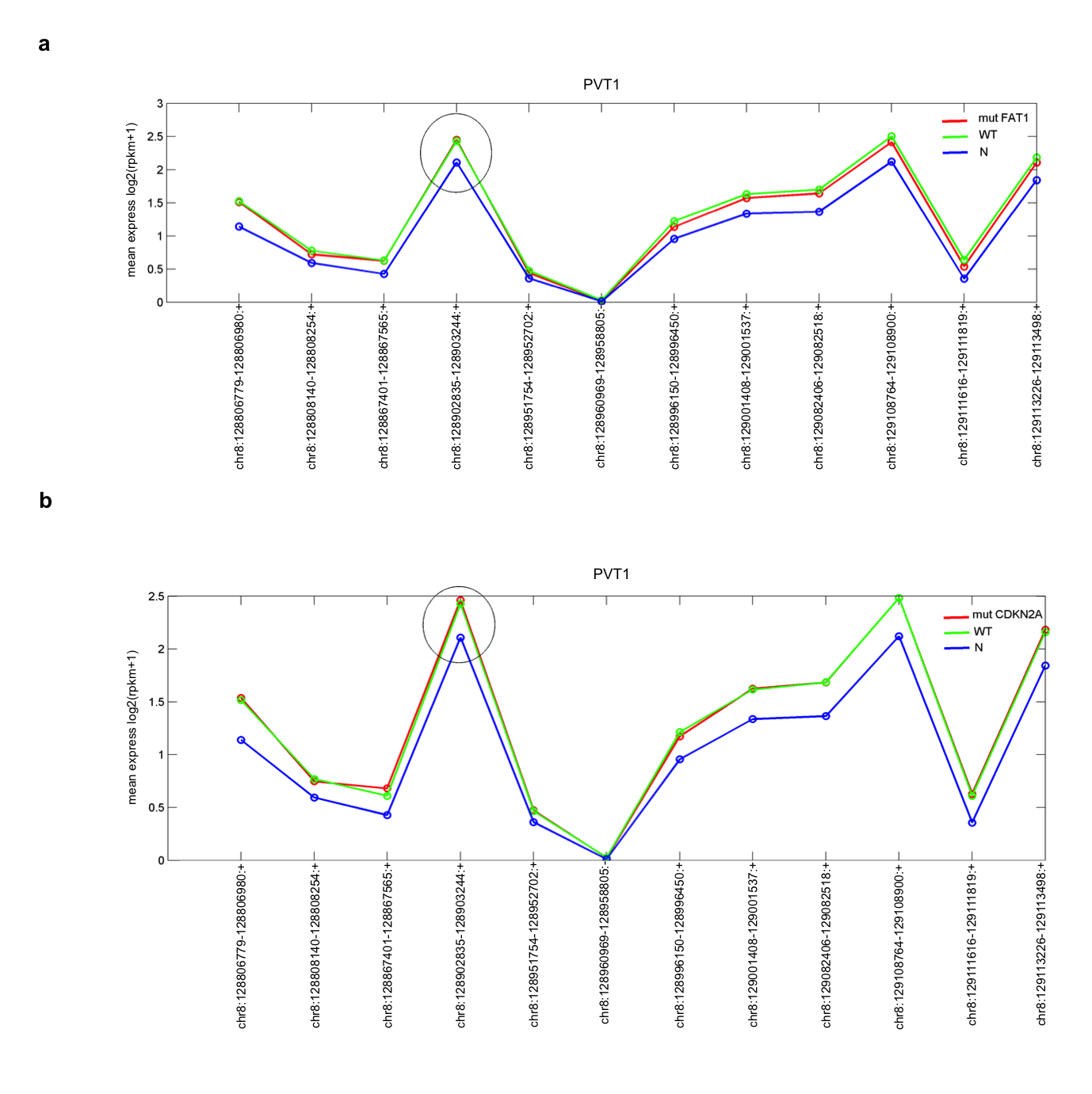
**Figure S1**

**Figure S1.** **Bioinformatic analysis of PVT1 in HNSCC cancer data set in relation to *FAT1* and *CDKN2A* mutations**

**a** Chromosomal intervals of PVT1 gene in tumor samples (divided in mutFAT1 and WT) and in non-tumoral samples. **b** Chromosomal intervals of PVT1 gene in tumor samples (divided in mutCDKN2A and WT) and in non-tumoral samples. The peak related to circPVT1 is indicated by a circle (Wilcoxon rank sum test, p < 0.05). (N, non-tumoral; WT, wild type).

**Figure S2**

**
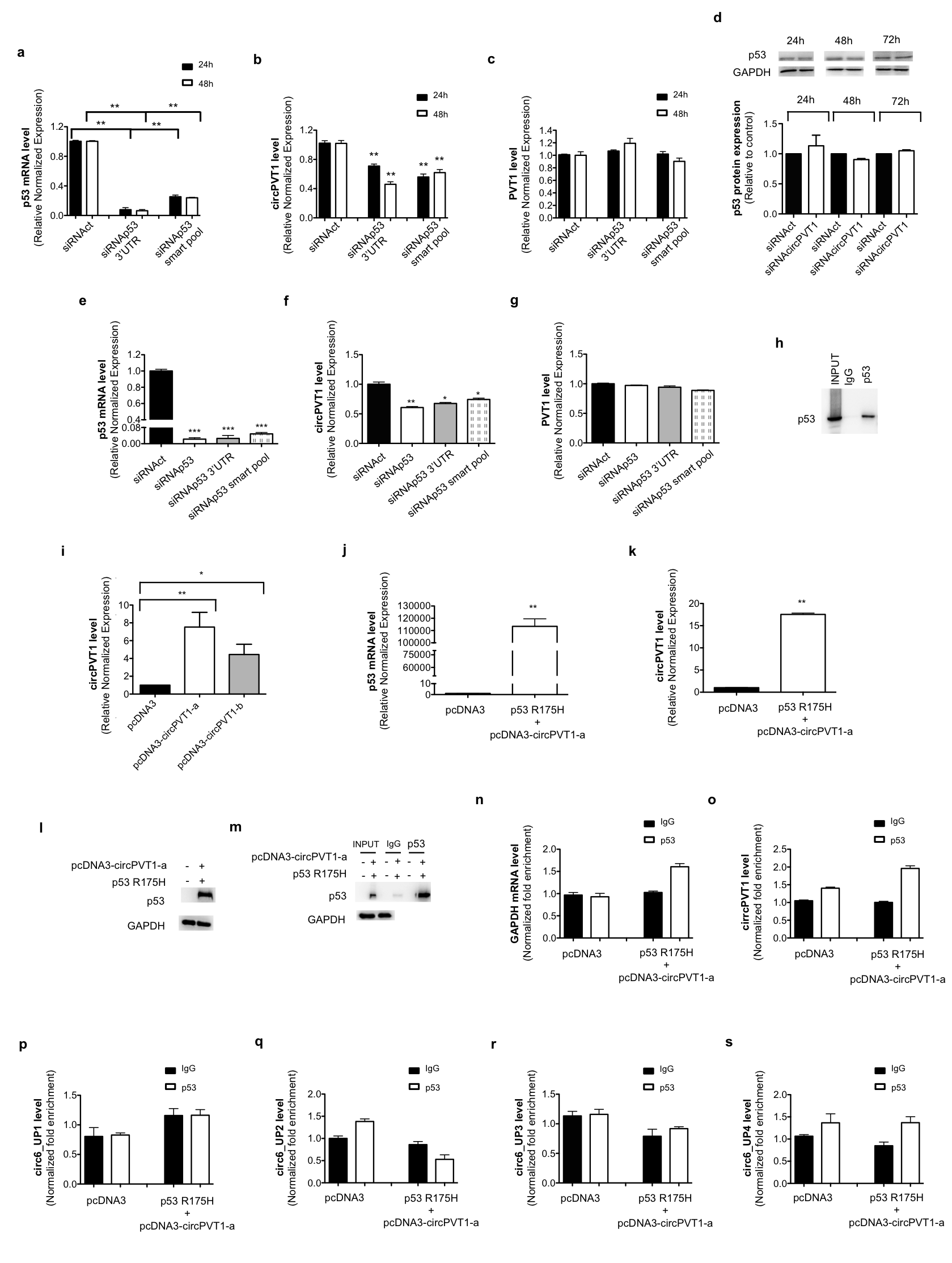
**

**Figure S2. mut-p53 affects the circPVT1 expression**

**a**-**d** CAL27 cell line. **a** p53 mRNA, **b** circPVT1 and **c** PVT1 level were detected by RT-qPCR in siRNAp53 3’UTR, siRNAp53 smart pool and in siRNAct. **d** p53 protein was detected by western blot in siRNAcircPVT1 and in siRNAct after 24, 48 and 72h from transfection. GAPDH was used as internal loading control. *Top*, a representative of the Western blot. *Bottom*, densitometric quantification of three independent experiments. Band intensity was normalized to that of GAPDH. **e**-**g** Detroit 562 cell line. **e** p53 mRNA, **f** circPVT1 and **g** PVT1 level were detected by RT-qPCR in siRNAp53, siRNAp53 3’UTR, siRNAp53 smart pool and in siRNAct after 48h from transfection. **h-i** CAL27 cell line. **h** p53 immunoprecipitation. **i** circPVT1 level was detected by RT-qPCR after transfection of the vectors pcDNA3-circPVT1-a and pcDNA3-circPVT1-b. We inserted the circPVT1 along with the endogenous flanking sequence into pcDNA3 obtaining the vector named pcDNA3-circPVT1-a. Afterward, we amplified part of the upstream flanking sequence and inserted it in an inverted orientation downstream, obtaining the vector named pcDNA3-circPVT1-b. **j-s** H1299 cell line. **j-k** p53 and circPVT1 levels were detected by RT-qPCR in pcDNA3 and in p53R175H+pcDNA-circPVT1-a. **l** p53 protein was detected by western blot in pcDNA-circPVT1-a and in p53R175H, GAPDH was used as internal loading control. **m** p53 immunoprecipitation in pcDNA3 and in p53R175H+pcDNA-circPVT1-a, GAPDH was used as internal loading control. **n-s** GAPDH, circPVT1, circPVT1_UP1, circPVT1_UP2, circPVT1_UP3, circPVT1_UP4 levels were analyzed by RT–qPCR. Normalization was performed to the amount of input RNA. Data are shown as mean of three replicates ± SD (Student’s test; *p < 0.05; **p < 0.01; ***p < 0.001).

**
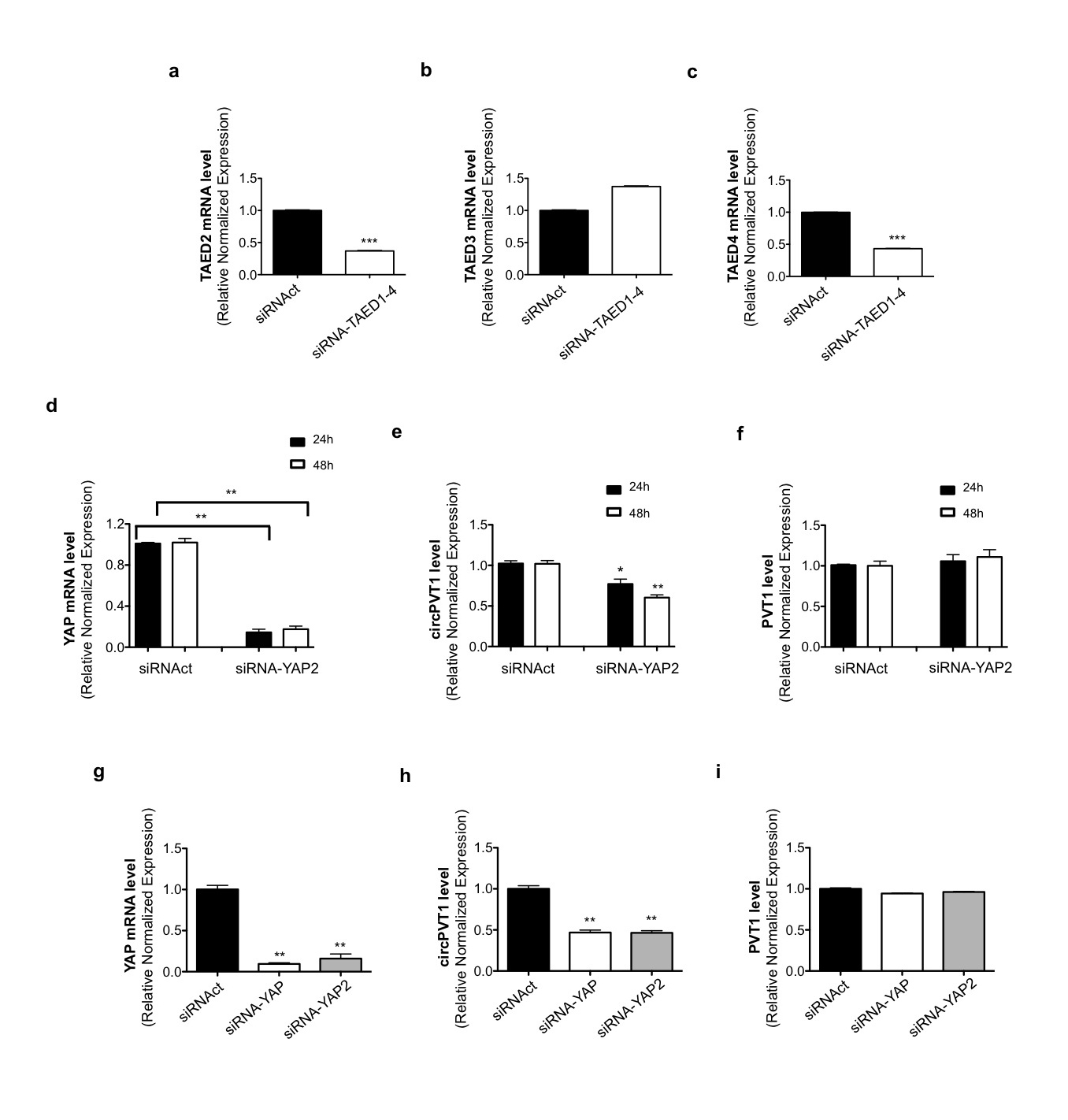
Figure S3**

**Figure S3. YAP affects the circPVT1 expression**

**a**-**f** CAL27 cell line. **a**-**c** TEAD2, TEAD3 and TEAD4 mRNA levels were detected by RT-qPCR in siRNA-TEAD1-4 and in siRNAct after 48h from transfection. **d** YAP mRNA level was detected by RT-qPCR in siRNA-YAP2 and in siRNAct. **e** circPVT1 level was detected by RT-qPCR in siRNA-YAP2 and in siRNAct. **f** PVT1 level was detected by RT-qPCR in siRNA-YAP2 and in siRNAct. **g**-**i** Detroit 562 cell line. **g** YAP mRNA level was detected by RT-qPCR in siRNA-YAP, siRNA-YAP2 and in siRNAct after 48h from transfection. **h** circPVT1 level was detected by RT-qPCR in siRNA-YAP, siRNA-YAP2 and in siRNAct after 48h from transfection. **i** PVT1 level was detected by RT-qPCR in siRNA-YAP, siRNA-YAP2 and in siRNAct after 48h from transfection. Data are shown as mean of three replicates ± SD (Student’s test; *p < 0.05; **p < 0.01; ***p < 0.001).

**
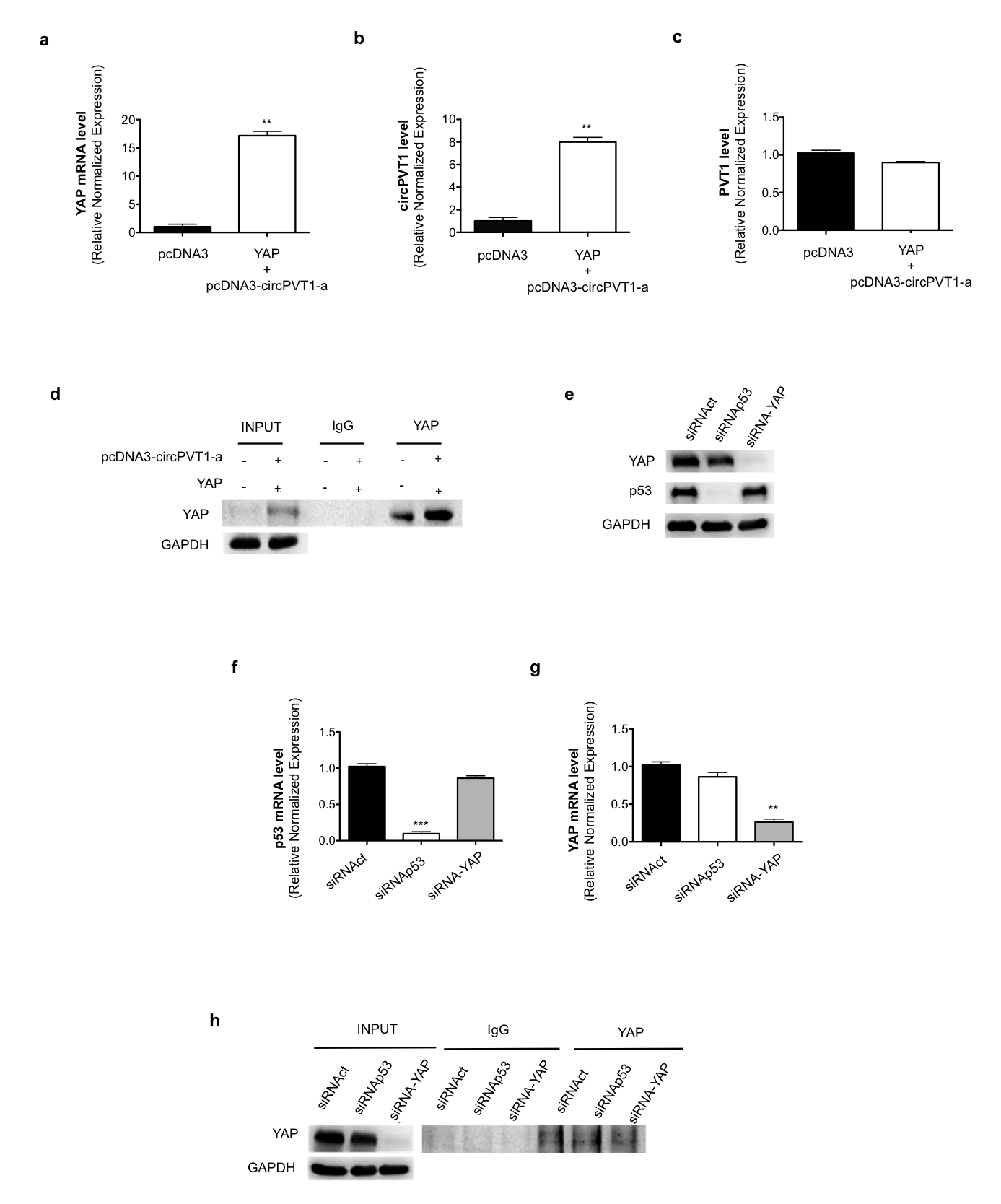
Figure S4**

**Figure S4. RIP analysis after transfection of YAP+pcDNA3-circPVT1a, siRNAp53 and siRNA-YAP in CAL27 cell line**

**a**-**c** YAP, circPVT1 and PVT1 levels were detected by RT-qPCR in pcDNA3 and YAP+pcDNA3-circPVT1a. **d** YAP immunoprecipitation in pcDNA3 and YAP+pcDNA3-circPVT1a, GAPDH was used as internal loading control. **e** YAP and p53 proteins were detected by western blot in siRNAp53 and in siRNA-YAP. GAPDH was used as internal loading control. **f**-**g** p53 and YAP mRNA levels were detected by RT-qPCR in siRNACt, siRNAp53 and siRNA-YAP. **h** YAP immunoprecipitation in siRNAct, siRNAp53 and siRNA-YAP, GAPDH was used as internal loading control. Data are shown as mean of three replicates ± SD (Student’s test; *p < 0.05; **p < 0.01; ***p < 0.001).

**
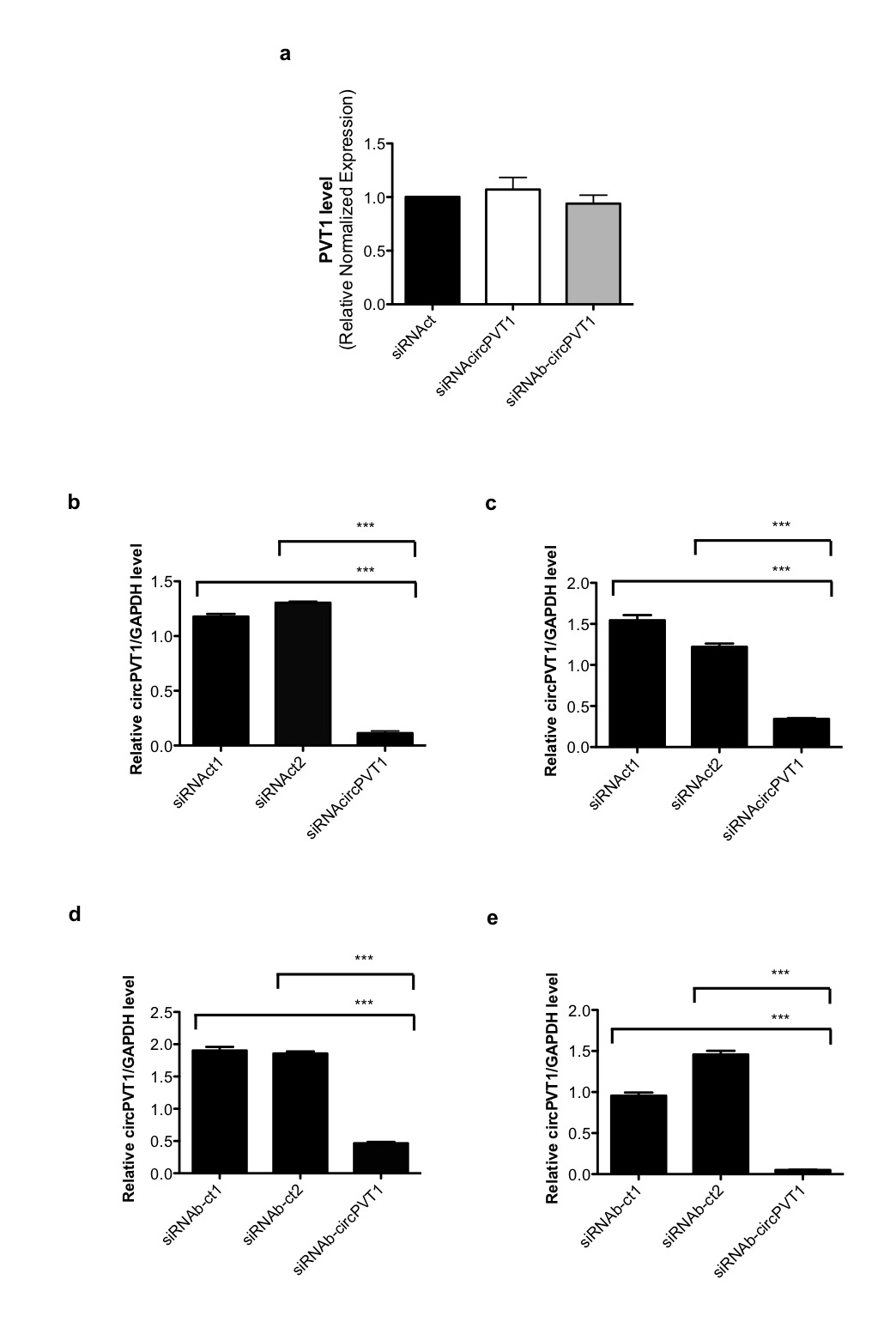
Figure S5**

**Figure S5. siRNAcircPVT1 and siRNAb-circPVT1 affect specifically the circPVT1 expression**

**a** PVT1 level was detected by RT-qPCR after transfection of siRNAcircPVT1 and siRNAb-circPVT1 in CAL27 cell line. **b** circPVT1 level was detected by RT-qPCR after transfection of siRNAct1, siRNAct2, and siRNAcircPVT1 in CAL27 cell line. **c** circPVT1 level was detected by RT-qPCR after transfection of siRNAct1, siRNAct2, and siRNAcircPVT1 in Detroit 562 cell line. **d** circPVT1 level was detected by RT-qPCR after transfection of siRNAb-ct1, siRNAb-ct2, and siRNAcircPVT1 in CAL27 cell line. **e** circPVT1 level was detected by RT-qPCR after transfection of siRNAb-ct1, siRNAb-ct2, and siRNAcircPVT1 in Detroit 562 cell line. Data are shown as mean of three replicates ± SD (Student’s test; *p < 0.05; **p < 0.01; ***p < 0.001).

**Figure S6**


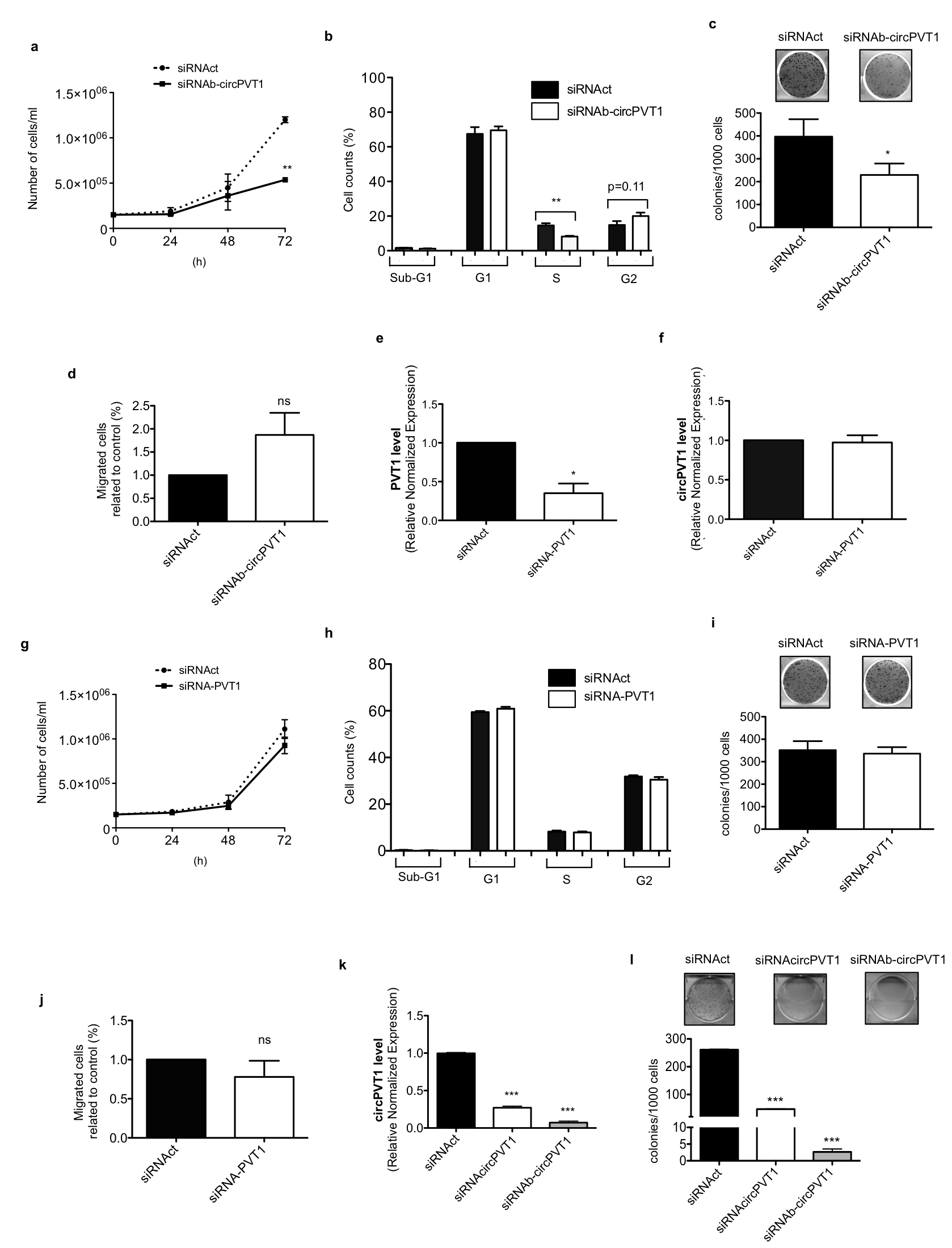


**Figure S6. CAL27 and FaDu phenotype after down-regulation of circPVT1 or PVT1**

**a**-**l** CAL27 cell line. **a** Cell proliferation was detected by cell counting after 24, 48 and 72h from transfection in siRNAb-circPVT1 and siRNAct. **b** Propidium iodide flow cytometric assay was performed to analyze the cell cycle in siRNAb-circPVT1 and siRNAct. **c** Colony formation assay was performed in siRNAb-circPVT1 and siRNAct. *Top*, a representative of colony formation assay. *Bottom*, quantification of three independent experiments by colony counting. **d** Migration assay was performed in siRNAb-circPVT1 and siRNAct**. e** PVT1 level was detected by RT-qPCR in siRNA-PVT1 and in siRNAct. **f** circPVT1 level was detected by RT-qPCR in siRNA-PVT1 and in siRNAct. **g** Cell proliferation was detected by cell counting after 24, 48 and 72h from transfection in siRNA-PVT1 and siRNAct. **h** Propidium iodide flow cytometric assay was performed to analyze the cell cycle in siRNA-PVT1 and siRNAct. **i** Colony formation assay was performed in siRNA-PVT1 and siRNAct. *Top*, a representative of colony formation assay. *Bottom*, quantification of three independent experiments by colony counting. **j** Migration assay was performed in siRNA-PVT1 and siRNAct. **k**-**l** FaDu cell line. **k** circPVT1 level was detected by RT-qPCR in siRNAcircPVT1, siRNAb-circPVT1 and in siRNAct. **l** Colony formation assay was performed in siRNAcircPVT1, siRNAb-circPVT1 and siRNAct. *Top*, a representative of colony formation assay. *Bottom*, quantification of three independent experiments by colony counting. Data are shown as mean of three replicates ± SD (Student’s test; ns, not significant; *p < 0.05; **p < 0.01; ***p < 0.001).


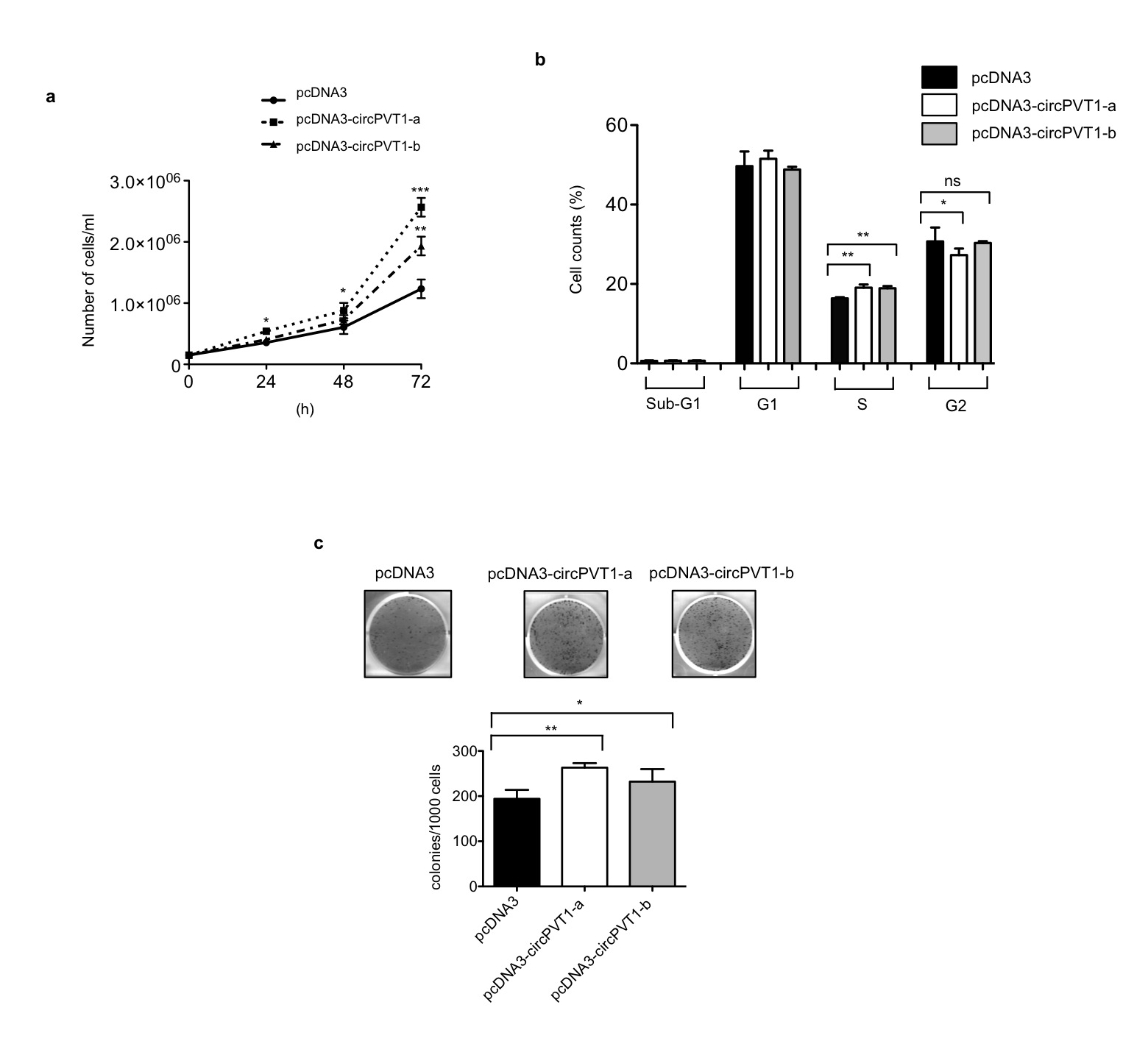
**Figure S7**

**Figure S7. CAL27 phenotype after over-expression of circPVT1**

**a** Cell proliferation was detected by cell counting after 24, 48 and 72h from transfection in pcDNA3-circPVT1-a, pcDNA3-circPVT1-b and pcDNA3. **b** Propidium iodide flow cytometric assay was performed to analyze the cell cycle in pcDNA3-circPVT1-a, pcDNA3-circPVT1-b and pcDNA3. **c** Colony formation assay was performed in pcDNA3-circPVT1-a, pcDNA3-circPVT1-b and pcDNA3. *Top*, a representative of colony formation assay. *Bottom*, quantification of three independent experiments by colony counting. Data are shown as mean of three replicates ± SD (Student’s test; ns, not significant; *p < 0.05; ** p < 0.01; ***p < 0.001).

**Figure S8**

**
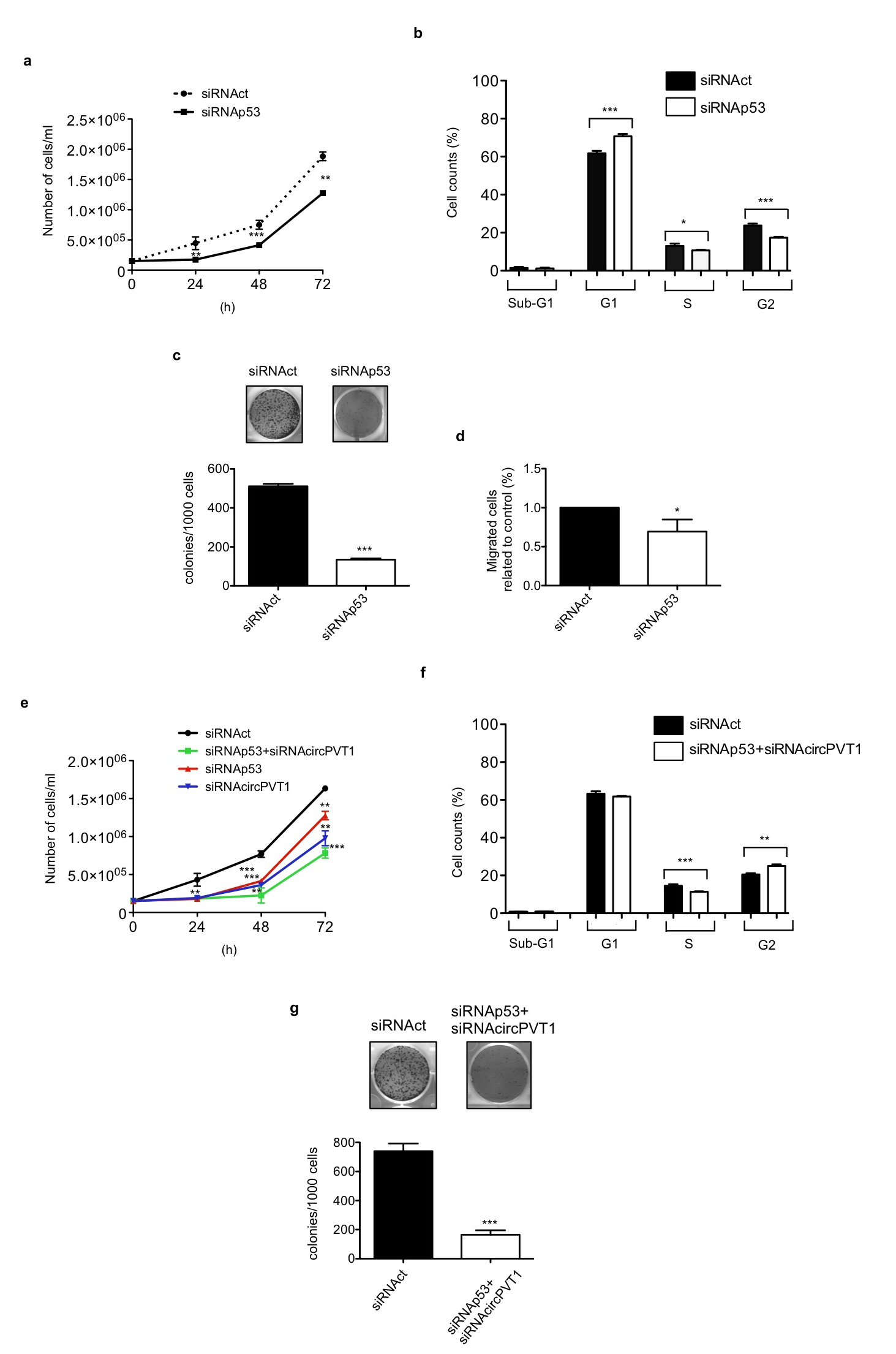
**

**Figure S8. CAL27 phenotype after down-regulation of p53 and after down-regulation of p53+siRNAcircPVT1**

**a** Cell proliferation was detected by cell counting after 24, 48 and 72h from transfection in siRNAp53 and in siRNAct. **b** Propidium iodide flow cytometric assay was performed to analyze the cell cycle in siRNAp53 and in siRNAct. **c** Colony formation assay was performed in siRNAp53 and in siRNAct*. Top*, a representative of colony formation assay. *Bottom*, quantification of three independent experiments by colony counting. **d** Migration assay was performed in siRNAp53 and in siRNAct. **e** Cell proliferation was detected by cell counting after 24, 48 and 72h from transfection in siRNAp53, siRNAp53+siRNAcircPVT1, siRNAcircPVT1 and in siRNAct. **f** Propidium iodide flow cytometric assay was performed to analyze the cell cycle in siRNAp53+siRNAcircPVT1 and siRNAct. **g** Colony formation assay was performed in siRNAp53+siRNAcircPVT1 and siRNAct. *Top*, a representative of colony formation assay. *Bottom*, quantification of three independent experiments by colony counting. Data are shown as mean of three replicates ± SD (Student’s test; *p < 0.05; ** p < 0.01; ***p < 0.001).


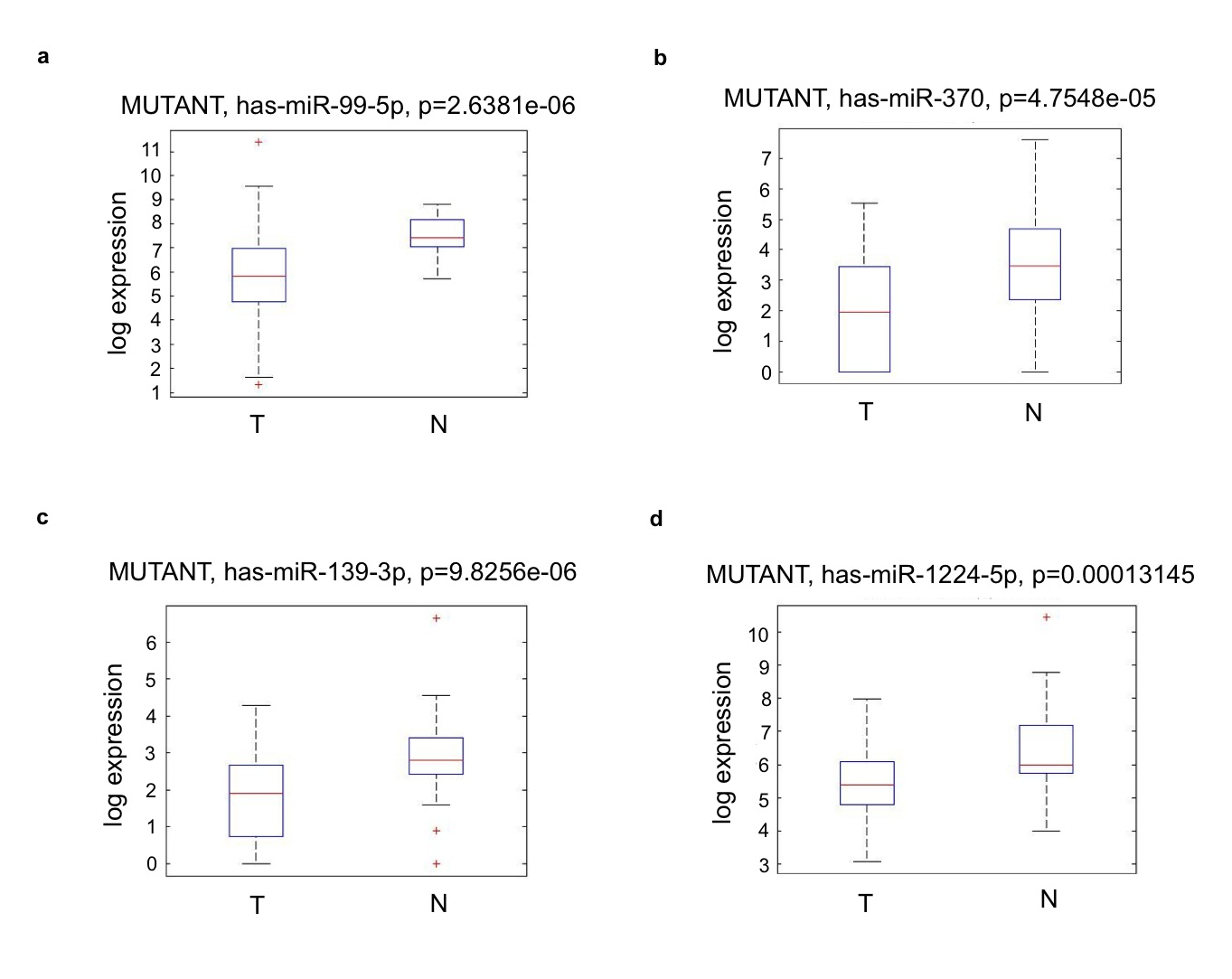
**Figure S9**

**Figure S9. Expression analysis of miRNAs p53-associated in HNSCC patients**

miRNAs expression analysis showed in [40] was used to evaluate the miRNAs expression in tumoral and normal samples. **a** miR-99-5p. **b** miR-370. **c** miR-139-3p. **d** miR-1224-5p. T, tumoral; N, normal.

**
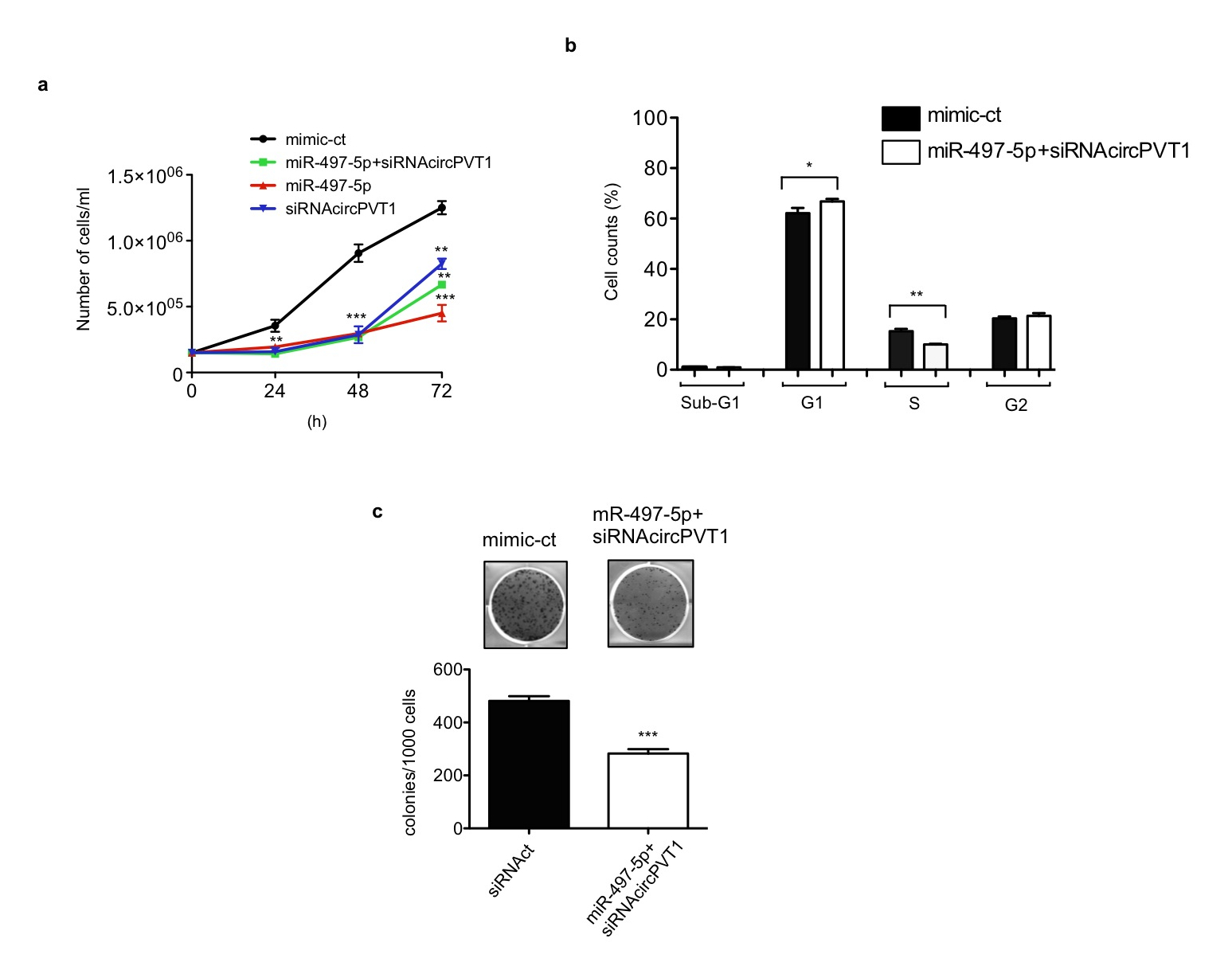
Figure S10**

**Figure S10. CAL27 phenotype after miRNA-497-5p up-regulation and circPVT1 down-regulation**

**a** Cell proliferation was detected by cell counting after 24, 48 and 72h from transfection in miR-497-5p, miR-497-5p+siRNAcircPVT1, siRNAcircPVT1 and in siRNAct. **b** Propidium iodide flow cytometric assay was performed to analyze the cell cycle in miR-497-5p, miR-497-5p+siRNAcircPVT1, siRNAcircPVT1 and in siRNAct. **c** Colony formation assay was performed in miR-497-5p+siRNAcircPVT1 and in siRNAct. *Top*, a representative of colony formation assay. *Bottom*, quantification of three independent experiments by colony counting. Data are shown as mean of three replicates ± SD (Student’s test; *p < 0.05; ** p < 0.01; ***p < 0.001).

**
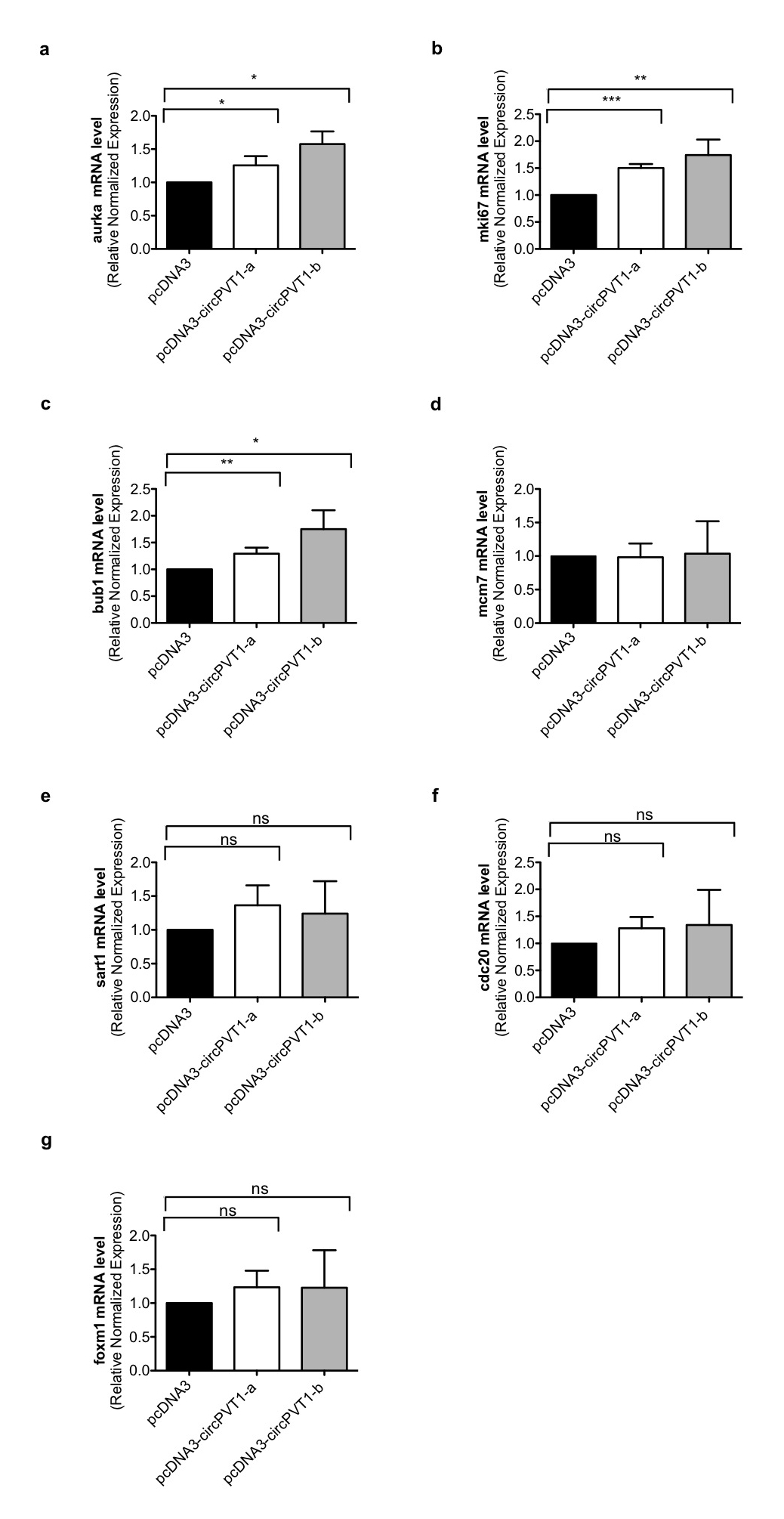
Figure S11**

**Figure S11. Expression analysis of selected genes after circPVT1 over-expression**

**a** aurka, **b** mki67, **c** bub1, **d** mcm7, **e** sart1, **f** cdc20, **g** foxm1 mRNA levels were detected by RT-qPCR in pcDNA3-circPVT1-a, pcDNA3-circPVT1-b and pcDNA3. Data are shown as mean of three replicates ± SD (Student’s test; ns, not significant; *p < 0.05; ** p < 0.01; ***p < 0.001).

**
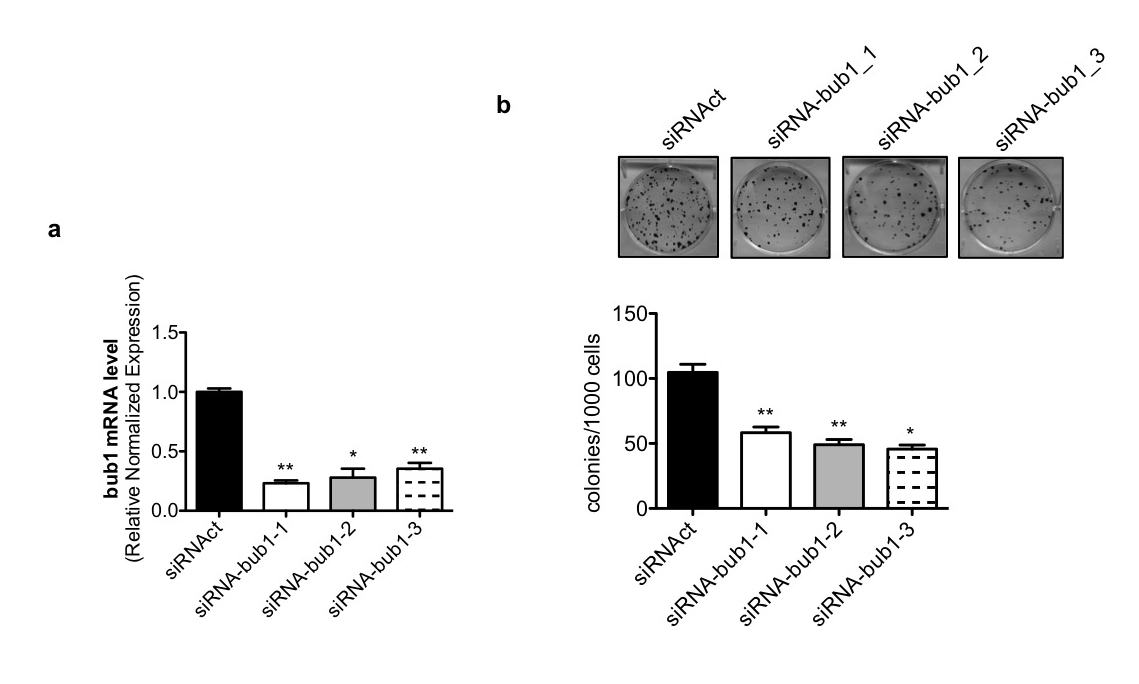
Figure S12**

**Figure S12. bub1 down-regulation affects the CAL27 phenotype**

**a** bub1 mRNA level was detected by RT-qPCR in siRNA-bub1-1, siRNA-bub1-2, siRNA-bub1-3 and siRNAct. **b** Colony formation assay was performed in siRNA-bub1-1, siRNA-bub1-2, siRNA-bub1-3 and siRNAct. *Top*, a representative of colony formation assay. *Bottom*, quantification of three independent experiments by colony counting. Data are shown as mean of three replicates ± SD (Student’s test; *p < 0.05; ** p < 0.01; ***p < 0.001).
